# Potential role of B- and NK-cells in the pathogenesis of pediatric aplastic anemia through deep phenotyping

**DOI:** 10.3389/fimmu.2024.1328175

**Published:** 2024-08-20

**Authors:** Lotte T. W. Vissers, Monique M. van Ostaijen-ten Dam, Janine E. Melsen, Yanna M. van der Spek, Koen P. Kemna, Arjan C. Lankester, Mirjam van der Burg, Alexander B. Mohseny

**Affiliations:** ^1^ Laboratory for Pediatric Immunology, Department of Pediatrics, Leiden University Medical Center, Willem Alexander Children’s Hospital, Leiden, Netherlands; ^2^ Department of Immunology, Leiden University Medical Center, Leiden, Netherlands; ^3^ Pediatric Hematology and Stem Cell Transplantation Unit, Department of Pediatrics, Leiden University Medical Center, Willem Alexander Children’s Hospital, Leiden, Netherlands

**Keywords:** aplastic anemia, pediatric, B-cells, natural killer cells, flow cytometry, bone marrow

## Abstract

**Introduction:**

Pediatric patients with unexplained bone marrow failure (BMF) are often categorized as aplastic anemia (AA). Based on the accepted hypothesis of an auto-immune mechanism underlying AA, immune suppressive therapy (IST) might be effective. However, due to the lack of diagnostic tools to identify immune AA and prognostic markers to predict IST response together with the unequaled curative potential of hematopoietic stem cell transplantation (HSCT), most pediatric severe AA patients are momentarily treated by HSCT if available. Although several studies indicate oligoclonal T-cells with cytotoxic activities towards the hematopoietic stem cells, increasing evidence points towards defective inhibitory mechanisms failing to inhibit auto-reactive T-cells.

**Methods:**

We aimed to investigate the role of NK- and B-cells in seven pediatric AA patients through a comprehensive analysis of paired bone marrow and peripheral blood samples with spectral flow cytometry in comparison to healthy age-matched bone marrow donors.

**Results:**

We observed a reduced absolute number of NK-cells in peripheral blood of AA patients with a skewed distribution towards CD56^bright^ NK-cells in a subgroup of patients. The enriched CD56^bright^ NK-cells had a lower expression of CD45RA and TIGIT and a higher expression of CD16, compared to healthy donors. Functional analysis revealed no differences in degranulation. However, IFN-γ production and perforin expression of NK-cells were reduced in the CD56^bright^-enriched patient group. The diminished NK-cell function in this subgroup might underly the auto-immunity. Importantly, NK-function of AA patients with reduced CD56^bright^ NK-cells was comparable to healthy donors. Also, B-cell counts were lower in AA patients. Subset analysis revealed a trend towards reduction of transitional B-cells in both absolute and relative numbers compared to healthy controls. As these cells were previously hypothesized as regulatory cells in AA, decreased numbers might be involved in defective inhibition of auto-reactive T-cells. Interestingly, even in patients with normal distribution of precursor B-cells, the transitional compartment was reduced, indicating partial differentiation failure from immature to transitional B-cells or a selective loss.

**Discussion:**

Our findings provide a base for future studies to unravel the role of transitional B-cells and CD56^bright^ NK-cells in larger cohorts of pediatric AA patients as diagnostic markers for immune AA and targets for therapeutic interventions.

## 1 Introduction

Bone marrow failure (BMF) in pediatric patients can be caused by several hematological disorders, including inherited bone marrow failure syndromes (IBMFS), (pre)malignant diseases and (idiopathic) aplastic anemia (AA). Independent of the etiology, hematopoietic stem cell transplantation (HSCT) is the most often used modality to cure severe BMF in pediatric patients. However, HSCT is an intensive medical procedure, associated with severe complications, such as graft versus host disease (GVHD), organ toxicity, secondary malignancies, and transplant-related mortality (1, 2). Therefore, it is essential to develop targeted therapies. A major limitation is the lack of diagnostic tools to identify the underlying event causing BMF in most pediatric patients suffering severe BMF.

In recent years, standard diagnostics for pediatric patients suspected of BMF have been expanded by wide genetic screening, telomere length analysis and broader immunological screening, resulting in the identification of a causative defect in 40% of the patients (3). The remaining group of BMF with unknown origin is classified as aplastic anemia (AA) (3). While the exact mechanism remains elusive, the prevailing hypothesis suggests that AA is driven by an immunological etiology. The evidence for an immune mechanism in these patients is the result of immunosuppressive therapy (IST) restoring blood counts in a part of AA patients (4). In addition, the identification of oligoclonal expanded T-cell population in experimental settings supports an immune-mediated pathophysiology (5). Several related mechanisms have been suggested, including CD8+CD57+ oligoclonal T-cells with direct cytotoxic activity (6), secretion of different inflammatory cytokines such as interferon-γ (IFN-γ) (7), immune imbalance by increased T-helper type 17 cells (8) or reduced regulatory T-cells (Tregs) (9), and associative correlations with certain HLA types (10).

The lack of a repeatedly identified type of effector cell, trigger or antigen causing immune AA in spite of decades of research suggests that complex disease mechanisms with involvement of multiple (immune) cell types and patient susceptibility to immune dysregulation are involved instead of a single disease causing cell or event. Recently, noncanonical activation of auto-reactive T-cells as effector cells attacking hematopoietic stem cells was shown in pediatric AA patients (11). However, the role of other important regulatory cells, such as NK- and B-cells, often hypothesized to be involved in adult patients, is understudied in pediatric patients (12, 13). In this study, we aimed to investigate the role of NK- and B-cells in pediatric AA patients suspected of an immune mediated disease causing mechanism through a comprehensive analysis of paired bone marrow and peripheral blood samples.

## 2 Methods

### 2.1 Patients

For this study, seven pediatric AA patients were included. The diagnosis of AA was based on the combination of peripheral cytopenia and hypocellular bone marrow and the exclusion of all other known causes of BMF by extensive diagnostics towards IBMFS, predisposition syndromes and secondary BMF as described previously (3). As controls, seven age-matched healthy bone marrow donors were included. Clinical data as well as paired peripheral blood (a volume of 4 to 10 mL for AA patients) and bone marrow samples were collected prior to treatment. The samples were processed by performing Ficoll density gradient centrifugation (LUMC Pharmacy, Leiden, The Netherlands) to isolate the PBMCs and BMMCs, respectively. The PBMCs and BMMCs were cryopreserved and used for phenotypic and functional analysis, with approval of the Institutional Review Board (protocols P00.068, P01.028, B17.001 and RP24.023) after informed consent was obtained.

For phenotypic and functional analysis, PBMCs from the same date were used, except for patient 4 (189 days difference) due to limited material availability. Patient 1 and healthy donor 5 were excluded from the functional analysis due to unavailability of materials. In addition, for AA patients, PBMCs and BMMCs were obtained within one month of each other to prevent large variabilities in cellular composition.

### 2.2 Phenotyping of peripheral blood and bone marrow mononuclear cells

PBMCs (up to 1 million viable cells) and BMMCs (up to 5 million viable cells) were stained with fluorochrome-conjugated antibodies. A two-tube flow cytometry panel was designed to study the lymphocytes in the peripheral blood. BMMCs were stained with 38 fluorochrome-conjugated antibodies in one tube (**Supplementary Table S1**).

The mononuclear cells were thawed using AIM-V medium (Thermo Fisher Scientific, Waltham, MA, USA) with 1% Penicillin/Streptomycin (Sigma-Aldrich, Saint Louis, MI, USA) and 20% heat inactivated fetal calf serum (FCS, Capricorn scientific, Ebsedorfgrund, Germany) (thawing medium) supplemented with 1600 IU per mL DNAse (VWR, Radnor, PA, USA), incubated for 5 minutes at 37°C, washed twice and incubated for one hour at 4°C (BMMC) or at 37°C (PBMC) in thawing medium. Viable cells were counted with a TC20 automated cell counter (Bio-Rad Laboratories, Hercules, CA, USA). The BMMCs were washed twice with PBS and stained with live dead staining (FVDUV455, eBioscience, San Diego, CA, USA) at 4°C in PBS. After washing twice in PBS supplemented with 0.5% Bovine Serum Albumin (BSA, Sigma-Aldrich), 2mM EDTA (Merck, Darmstadt, Germany) and 0.02% NaN_3_ (LUMC Pharmacy) (FACS buffer), PBMC and BMMC samples were incubated for 45 minutes at room temperature with the fluorochrome-conjugated antibodies for extracellular markers and Brilliant Stain buffer plus (Becton Dickinson Biosciences (BD), Franklin Lanes, NY, USA) in FACS buffer. PBMC samples were washed three times with FACS buffer and kept in the fridge until measurement. BMMC samples were washed twice in PBS/NaN_3_, and subsequently cells were fixed in 4% paraformaldehyde in PBS and permeabilized in PBS supplemented with 0.5% BSA, 2mM EDTA, 0.02% NaN_3_ and 0.1% saponin (Perm buffer). After 10 minutes incubation with 10% FCS in Perm buffer at 4°C and subsequent washing of cells with Perm buffer, antibodies for intracellular staining diluted in Perm buffer were added and incubated for 30 minutes at 4°C. Next, the cells were washed three times, resuspended in Perm buffer and kept in the fridge until measurement. DAPI was added to the PBMC samples prior to measurement to detect dead cells. Data was acquired on the 5-Laser Cytek Aurora flow cytometer (Cytek^®^ Biosciences Inc, Fremont, CA, USA) at the Flow cytometry Core Facility (FCF) of the Leiden University Medical Center (LUMC) using Spectroflo^®^ Software (Cytek Biosciences Inc).

### 2.3 *In vitro* assessment of NK-cell degranulation and IFN-γ production

To study degranulation and cytokine production of NK-cells, PBMCs were thawed as described and plated in a flat-bottom 48 wells plate (Corning Incorporated, Corning, NY, USA) at a concentration of 5 million per mL in AIM-V supplemented with 1% Penicillin/Streptomycin, 1% glutamax and 10% heat inactivated FCS and incubated at 37°C with 5% CO_2_ in a 100% humidified atmosphere. The next day, CD107a (BD, FITC) was added. Subsequently, K562 target cells (1:1 effector-target cell ratio) or a combination of 10 ng/mL IL-12 (PeproTech, Rocky Hill, NJ, USA), 10 ng/mL IL-15 (PeproTech) and 20 ng/mL IL-18 (MBL International, Woburn, MA, USA) were added, or cells were incubated in medium only, as control. After 1 hour of incubation, GolgiStop (BD) was added, followed by another 3 hours of incubation. The stimulated PBMCs were harvested and stained for surface markers (**Supplementary Table S1**). Subsequently, cells were fixed and permeabilized as described, incubated with Fc block (eBioscience) for 10 minutes at 4°C and stained intracellularly (**Supplementary Table S1**). Data was acquired on the 3-Laser Cytek Aurora flow cytometer (Cytek^®^ Biosciences Inc) at the FCF of LUMC using Spectroflo^®^ Software.

### 2.4 Cytometry data analysis

Flow cytometry data analysis was performed using the OMIQ data science platform (Omiq, Inc, Santa Clara, CA, USA). Manual compensation and arcsinh transformation was performed, as described previously (14). FlowAI was used to remove anomalous events, based on flow rate and outlier events (15). Data was normalized using CytoNorm (16) based on a reference control. Cell populations were identified using the gating strategy described in **Supplementary Figures S1**-**S3**. Absolute blood cell counts were calculated using the leukocyte subsets (absolute counts and differential) which were determined on an automated hematology analyzer.

For single-cell analysis, UMAP was performed as dimensionality reduction (17), and FlowSOM (18) for clustering. Calculations were based on all normalized expression values in the respective panel, except the live/dead marker, CD10, CD15, CD38 and IgD. Peripheral blood NK-cells were defined as Lin^-^CD56^+^CD7^+^ (**Supplementary Figure S2**) and downsampled to include an equal number per group (e.g., healthy donors and AA patients). Bone marrow derived B- and progenitor cells were defined as described in **Supplementary Figure S1** and also downsampled to include an equal number per group.

### 2.5 Statistical analysis

Visualizations and statistics were performed in GraphPad Prism v9.3.1. (GraphPad Software, San Diego, CA, USA). Heatmaps were generated using the pheatmap package in R (v4.2.2, R Foundations for Statistical Computing, Vienna, Austria). The Mann-Whitney U test was applied for the analysis of non-parametric data. Fisher’s exact test was used for the analysis of categorical data. A two-sided p-value of less than 0.05, corrected for false discovery rate (FDR), was considered statistically significant.

## 3 Results

### 3.1 Patient characteristics

In this study, seven pediatric AA patients and seven age-matched healthy donors were included (**Table 1**; **Supplementary Table S2**). No significant differences in age at sampling and sex were found between both groups (p=0.71 and 0.56, respectively). Five out of seven patients had classic (very) severe AA according to the WHO classification with hypocellular bone marrow and trilinear severe cytopenia. Patient 4 presented with progressive peripheral cytopenia and marrow hypoplasia 10 years after HSCT for very severe AA and later she also developed paroxysmal nocturnal hemoglobinuria (PNH). This patient was initially transplanted at the age of 4 years by using a HLA-identical sibling. The procedure was without complications and at yearly follow-up she was free of symptoms for 10 years. During the recurrent presentation with cytopenia 10 years post-HSCT, she underwent extensive diagnostic work-up (DNA was isolated from fibroblasts cultured from skin to avoid donor DNA contamination) which revealed no explanation for the peripheral cytopenia and progressive marrow hypoplasia.

**Table 1:** Patient characteristics.

Patient	Gender	Age at diagnosis (y)	Age at sampling (y)	PNH clones	TPO IU/mL	Auto antibodies*	Viral screen **	Deficiencies***	Bone marrow cellularity	Bone marrow hematopoiesis	Bone marrow clonality	Pathogenic genetic variation for BMF	Other genetic variation	MMC-test	Telomere length	CMV status
**1**	f	15	16	Ery<1%, Neu 7%, Mono 16%	429	No	Neg	No	Hypocellular	Decreased hematopoiesis in all lines, increased lymphocytes and mast cells	No (also no monosomy 7)	No	VUS SAMD9L;Chr7(GRCh37): g.92764011T>A:NM_152703.2:c.1274A>T p.(His425Leu) Heterozygous	Normal	Normal	Pos
**2**	m	8	9	<1%	151	No	Neg	No	Hypocellular	Decreased hematopoiesis in all lines, increased mast cells	No	No	VUS RPS24;Chr10(GRCh37): g.79795104T>A:NM_001142285.1:c.4-6T>A p.()? Heterozygous	Normal	Normal	Pos
**3**	m	2	3	<1%	714	HLA class I	Neg	No	Hypocellular	Minimal rest hematopoiesis without dysplasia	No (also no monosomy 7)	No	VUS SAMD9;Chr7(GRCh37): g.92733281T>G:NM_017654.3:c.2130A>C p.(Lys710Asn) Heterozygous	Normal	P1 in all fractions	Pos
**4**	f	16	18	Ery 20-30%, other fractions 50%	209	No	Neg	No	Progressive hypocellular	Decreased myelopoiesis and megakaryopoiesis with relative increased erythropoiesis	No	No	No	NE	NE	Neg
**5**	m	5	5	<1%	293	No	Neg	No	Hypocellular	Minimal rest hematopoiesis without dysplasia	No	No	BRCA1;Chr17(GRCh37): g.41234520G>A:NM_007294.3:c.4258C>T p.(Gln1420*) Heterozygous	Normal	Granulocytes <p1	Neg
**6**	m	16	17	<1%	476	No	Neg	No	Hypocellular	Decreased hematopoiesis in all lines, no dysplasia	No	No	No	Normal	Granulocytes <p1	Pos
**7**	f	3	3	<1%	234	No	Neg	No	Hypocellular	Decreased hematopoiesis in all lines, absent megakaryocytes, increased B- an T- cells	No	No	No	Normal	Normal	Pos

* Anti-nuclear antibodies (ANA), direct antiglobulin test (DAT) and human leukocyte antigen (HLA) antibodies were screened, followed by further specification if tested positive.

** Screened for active ParvoB19, EBV, CMV, Hepatitis A/B/C (if indicated Leishmania and HIV).

*** Iron, folic acid, vitamin B12, thyroid/liver/kidney function, electrolytes.

PNH, paroxysmal nocturnal hemoglobinuria; TPO, thrombopoietin; BMF, bone marrow failure; MMC, mitomycin C; CMV, cytomegalovirus; VUS, variant of unknown significance; NE, not examined. The symbol ? indicates that the effect of the nucleotide change on the resulting protein is not clear.

Although for this study only patients were included without IBMFS or other identified cause for BMF, whole exome sequencing (WES) data revealed interesting variants which were classified as variants of unknown significance (VUS) in three out of seven patients. In addition, in one patient, a pathogenic variant BRCA1, which is not associated with BMF, was found. Mitomycin C (MMC) DNA breakage test results were normal for all patients. Patients 5 and 6 had shortened telomere length in the granulocyte fraction, patient 3 had short telomere length for all fractions, however not to the extent that telomere biology disorder (TBD) was suspected. All other patients had normal telomere length. Telomere length for patient 4 could not be performed, due to high donor chimerism in peripheral leukocytes.

### 3.2 Absolute B- and NK-cell counts in peripheral blood of AA patients are reduced

**Figure 1:**
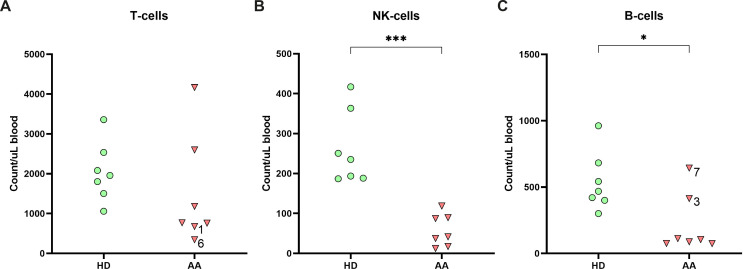
Lymphocyte distribution in peripheral blood. Absolute numbers of **(A)** T-cells, **(B)** NK-cells and **(C)** B-cells in the peripheral blood of AA patients compared to age-matched healthy donors (HD). Data were compared using the Mann-Whitney U test. Significant values after multiple testing correction using the false discovery rate (FDR) are indicated: ***P<0.001; *P<0.05.

To study the lymphocyte composition in AA patients, cryopreserved PBMCs were analyzed by flow cytometry. No significant differences in the absolute number of T-cells were found between AA patients and healthy donors (**Figure 1A**). However, in two AA patients (1 and 6) the T-cell number was below the normal range of age-matched controls (**Supplementary Table S3**). In contrast, absolute NK-cell and B-cells were significantly lower in AA patients as compared to healthy controls (**Figures 1B, C**). Six out of seven AA patients exhibited NK-values below the lower limit of age-matched controls (**Supplementary Table S3**) (19). The seventh patient displayed markedly reduced NK-cell numbers, just within the normal range. Regarding the B-cells, four patients had B-cells numbers below and one just within the normal range. The lymphocyte subsets from the healthy donors were all within normal range.

### 3.3 CD56^bright^ NK-cells with a non-classical phenotype are enriched in peripheral blood of a subgroup of AA patients

**Figure 2:**
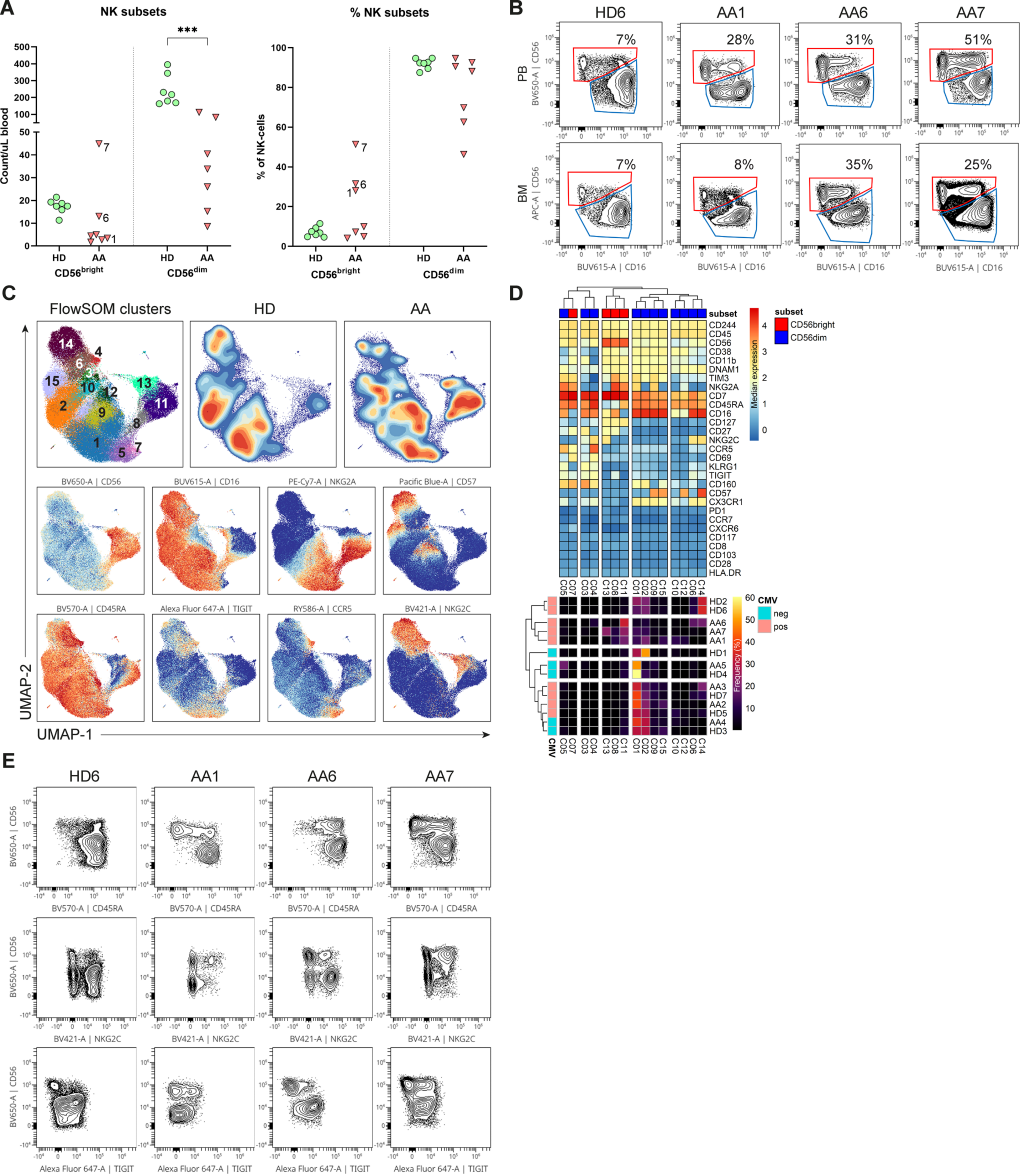
NK-cells in blood and bone marrow. **(A)** Absolute and relative CD56^dim^ and CD56^bright^ NK-cell values within the blood of AA patients and healthy donors (HD). NK-cells were defined as Lin^-^CD56^+^CD7^+^. **(B)** Representative flow cytometry plots of total NK-cells from one healthy donor and 3 AA patients with enriched CD56^bright^ NK-cells. The CD69^+^ tissue-resident NK-cells were excluded from the total NK-cell population in bone marrow. **(C)** UMAP and FlowSOM clustering of total blood NK-cells based on all fluorochrome conjugated markers, except the live/dead marker. An equal number of cells per group (HD vs AA) was included in the analysis, resulting in a minimum of 5.533 and a maximum of 9.449 events per sample. **(D)** A heatmap based on median expression levels and proportions of each individual cluster. **(E)** Flow cytometry plots demonstrating expression of CD45RA, TIGIT and NKG2C in a subgroup of AA patients with enriched CD56^bright^ NK-cells. One representative healthy donor is shown. Data were compared using the Mann-Whitney U test. Significant values after multiple testing correction using the false discovery rate (FDR) are indicated: ***P<0.001; *P<0.05.

To study whether the NK-cells in AA patients have an aberrant phenotype, we performed an in-depth single-cell analysis of the flow cytometry data. First, NK-cells were categorized into the conventional CD56^dim^CD16^+^ and CD56^bright^CD16^+/-^ NK-cell subset. Although the absolute number of CD56^dim^ NK-cells in blood was consistently lower compared to healthy donors in all patients, the CD56^bright^ NK-cells were relatively enriched or even higher in absolute counts in 3 AA patients (1, 6 and 7, **Figures 2A, B**). Within the bone marrow, a similar enrichment of CD56^bright^ NK-cells was observed (**Figure 2B**).

Next, dimensionality reduction (UMAP) and FlowSOM clustering were performed on the blood NK-cells (**Figure 2C**; **Supplementary Figure S4**). In total 15 clusters were identified, each representing a unique phenotype (**Table 2**). Based on the density of the UMAP, it was evident that the distribution of the clusters differed between healthy donors and AA patients (**Figure 2C**). In line with our results as described above, cluster 8, 11 and 13 representing CD56^bright^ NK-cells were abundant in AA patients 1, 6 and 7 (**Figures 2C, D**). Cluster 11 was characterized by a classical CD56^bright^ phenotype NKG2A^+^CD45RA^+^CD16^-^. Notably, cluster 8 was uniquely present in patient 1 (9.4% of NK-cells) and patient 7 (8.5% of NK-cells) and was characterized by a CD45RA^-/dim^TIGIT^+^CD16^+^ phenotype. Cluster 13, representing 22.5% of NK-cells in patient 7, included CD45RA^-/dim^NKG2C^+^ cells (**Figures 2D, E**). Within the CD56^dim^ compartment, only notable differences in NKG2C^+^ percentages were detected, but this could be attributed to CMV serostatus as previously described (**Figure 2D**) (20–22). In conclusion, the CD56^bright^ NK-cells that are enriched in a subgroup of AA patients have an aberrant phenotype.

**Table 2:** Definition of NK-clusters.

Cluster	Phenotype
1	CD56^dim^NKG2A^+^
2	CD56^dim^NKG2A^-^CD57^-^
3	CD56^dim^NKG2A^-^CD27^+^CD160^hi^
4	CD56^dim^NKG2A^-^NKG2C^+^CCR5^+^TIGIT^hi^
5	CD56^intermediate^NKG2A^+^CCR5^+^TIGIT^hi^
6	CD56^dim^NKG2A^-^CD57^-^NKG2C^+^
7	CD56^bright^NKG2A^+^CD27^+^CD69^+^CD160^hi^
8	CD56^bright^NKG2A^+^CD27^+^CD16^+^TIGIT^+^CD45RA^-^
9	CD56^dim^NKG2A^+^CD57^+^
10	CD56^dim^NKG2A^-^CD57^-^NKG2C^-^CD16^dim^
11	CD56^bright^NKG2A^+^
12	CD56^dim^NKG2A^-^CD57^+^CD16^dim^
13	CD56^bright^NKG2A^dim^NKG2C^+^CD45RA^-^
14	CD56^dim^NKG2A^-^NKG2C^+^CD57^+^
15	CD56^dim^NKG2A^-^CD57^+^

### 3.4 NK-cell function is reduced in the CD56^bright^-enriched patient group

**Figure 3:**
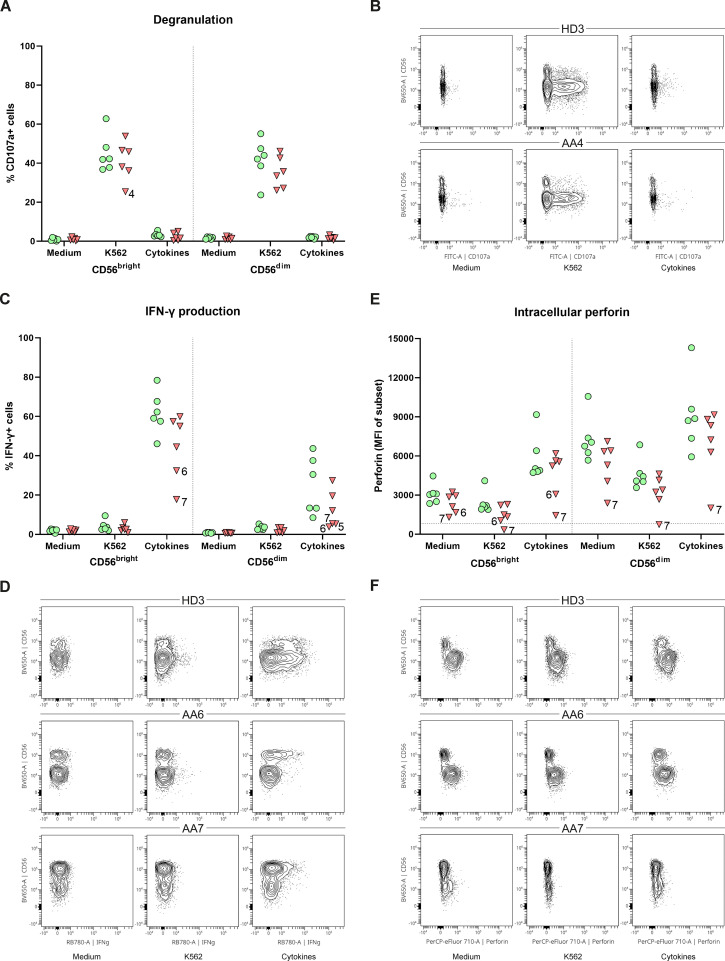
Function of peripheral NK-cells. NK-cells were stimulated with medium, K562 cells or a combination of cytokines (IL-12, IL-15, IL-18). **(A)** Degranulation of NK-cell subsets. **(B)** Representative flow cytometry plots demonstrating a reduction of CD107a^+^CD56^bright^ in patient 4. One representative healthy donor (HD) is shown. **(C)** Percentage of IFN-γ positive cells for both CD56^dim^ and CD56^bright^ NK-cell subsets. **(D)** Representative flow plots of one HD and two AA patients with reduced IFN-γ production of both CD56^dim^ and CD56^bright^ NK-cell subsets. **(E)** Intracellular expression of perforin of both CD56^dim^ and CD56^bright^ NK-cell subsets indicated by the mean fluorescence intensity (MFI). Horizontal dotted line visualizes the geomean of perforin negative T-cells (negative control). **(F)** Representative flow cytometry plots showing reduced perforin expression of CD56^bright^ NK-cell in AA6 and reduced expression of both subsets in AA7. One representative (HD) is shown. Data were compared using the Mann-Whitney U test. After multiple testing correction using the false discovery rate (FDR) no significant differences were observed.

To study the link between the CD56^bright^ expansion and AA, functional NK-cell assays were performed. PBMCs were stimulated with K562 cells, cytokines or, as a control with medium. The degranulation of CD56^bright^ and CD56^dim^ NK-cells, as assessed by CD107a expression, did not significantly differ between AA patients and healthy donors (**Figure 3A**). Only patient 4 had less externalization of CD107a on CD56^bright^ NK-cells after stimulation with K562 cells (25.4%) compared to healthy controls (mean 44.9%) (**Figures 3A, B**). IFN-γ production by CD56^bright^ NK-cells of both patient 6 (32.4%) and patient 7 (17.8%) was lower compared to healthy donors (mean 61.8%) upon stimulation with cytokines (**Figures 3C, D**). Although less apparent, IFN-γ production by CD56^dim^ NK-cells seemed hampered in patient 5 (5.4%), 6 (3.8%) and 7 (5.5%) compared to healthy donors (mean 24.5%) (**Figures 3C, D**). Interestingly, in all conditions, patient 6 and 7 had reduced perforin expression of CD56^bright^ NK-cells compared to healthy donors. This reduction became most pronounced upon stimulation with cytokines, with a mean fluorescence intensity (MFI) of 3071 and 1148 for patient 6 and 7, respectively, compared to a MFI of 5655 (geomean) for the healthy donors (**Figures 3E, F**). For patient 7, a similar pattern was seen for the CD56^dim^ NK-cells. Together, these results suggest that NK-cell function is altered in the CD56^bright^-enriched patient group.

### 3.5 Transitional B-cells are decreased in AA patients

**Figure 4:**
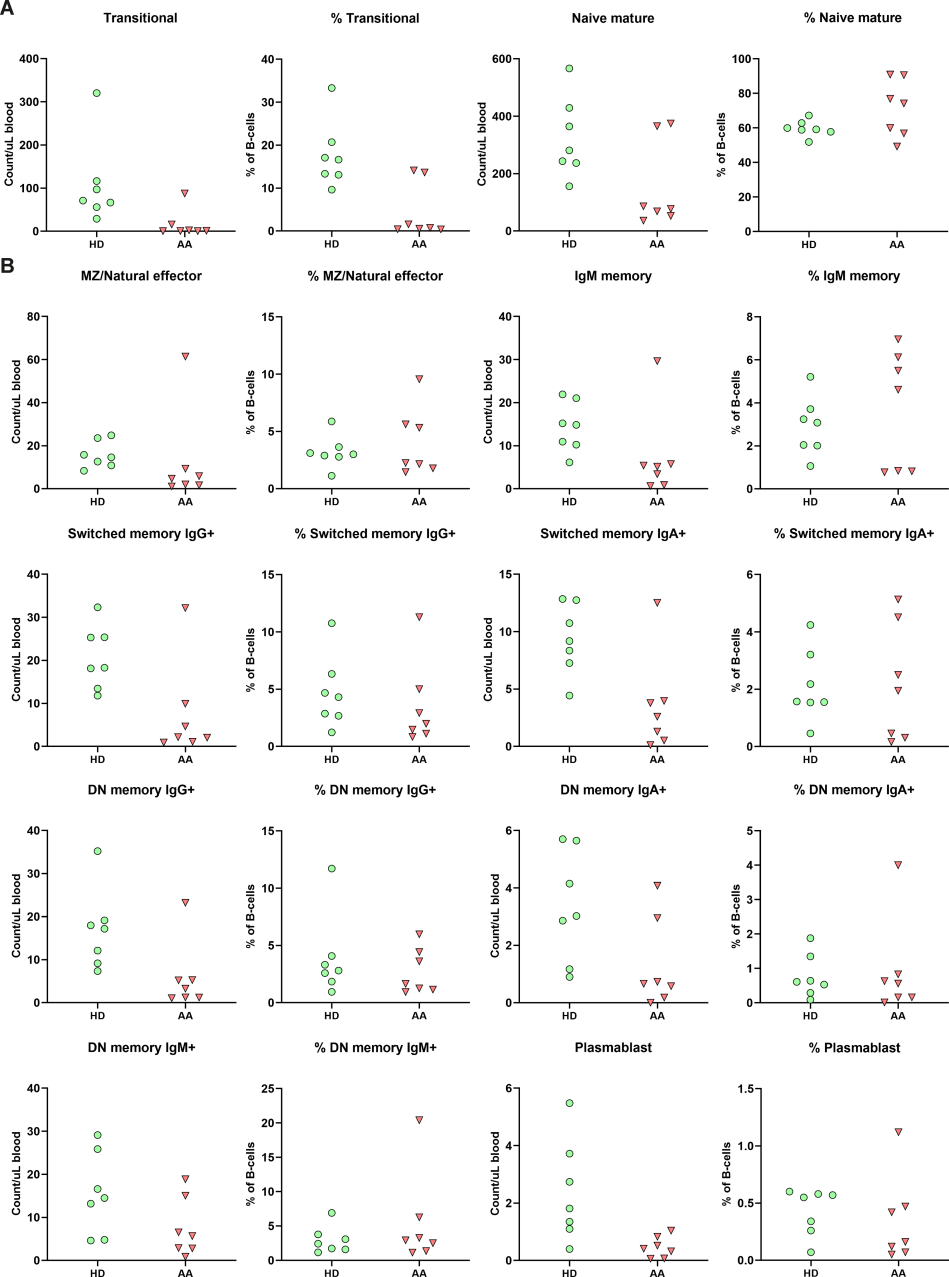
B-cell subpopulation within the peripheral blood. **(A)** Relative and absolute numbers of transitional and naïve mature B-cells in AA patients compared to healthy donors (HD). **(B)** Relative and absolute number of natural effector, memory B-cells and plasmablasts. Data were compared using the Mann-Whitney U test. After multiple testing correction using the false discovery rate (FDR) no significant differences were observed. DN, double-negative; MZ, marginal zone.

As AA patients had reduced absolute B-cell values in peripheral blood, the composition was studied in further detail. While no significant differences after multiple test correction were observed, subset analysis indicated that the absolute numbers of B-cell subsets of nearly all SAA patients were reduced compared to the donor controls (**Figure 4**). Interestingly, although most mature B-cells, including the switched memory B-cells, were reduced in number, Ig production was not affected (**Supplementary Figure S5**), indicating that the B-cell immune response in AA patients is still intact.

The transitional B-cells of AA patients were the only subset with a trend towards reduction in both absolute numbers and relative values compared to the donors (**Figure 4**). When comparing to age-specific references, five of these patients exhibited absolute numbers below the normal range (**Supplementary Table S3**) (23).

### 3.6 Absence of transitional B-cells seems independent of bone marrow precursor B-cell development

As transitional B-cells are early emigrant cells from the bone marrow, flow cytometry analysis was performed on BMMCs to determine whether these abnormal cell counts were due to aberrant precursor B-cell development. The bone marrow composition revealed significantly decreased percentages of progenitor cells, myeloid cells, erythroid cells, B-cells and significantly increased percentages of T- and NK-cells (**Supplementary Figure S6**). However, higher levels of blood contamination due to hypoplastic bone marrow in AA patients might partly explain the increased T- and NK cell percentages (**Supplementary Table S4**).

**Figure 5:**
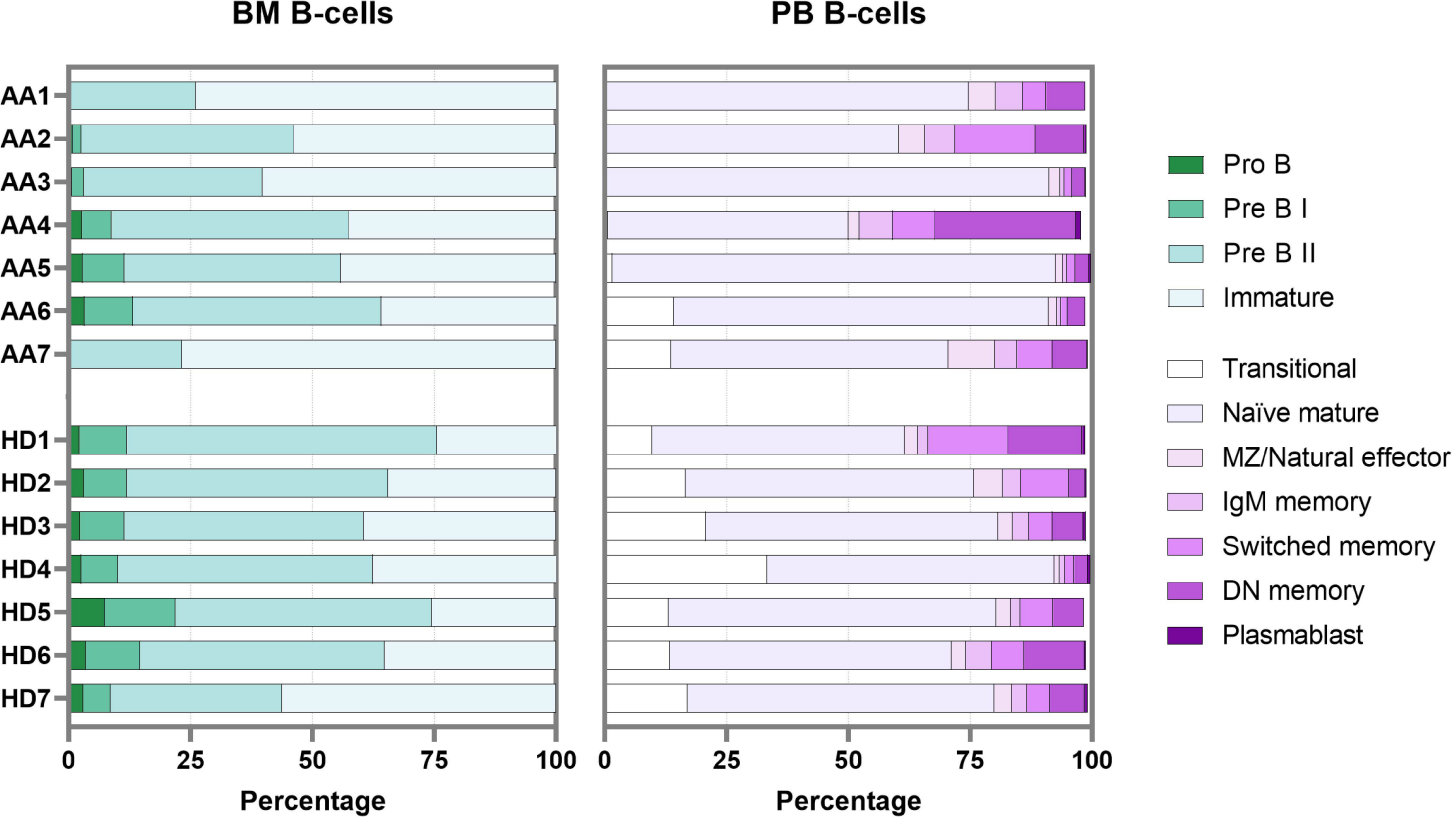
B-cell development in bone marrow and peripheral blood. Each bar represents one individual. BM, bone marrow; PB, peripheral blood; DN, double-negative; MZ, marginal zone.

To overcome the limitation of blood contamination, only B-cells known to be exclusively present in the bone marrow were analyzed first (precursor B-cells). These included pro B, pre B I, pre B II and immature B-cells. In general, significant lower pre B I, pre B II and significantly higher immature B-cells were observed in patients (**Supplementary Figure S7**). Although absolute counts in AA patients were likely further skewed due to their hypocellular bone marrow, there was no block in precursor B-cell development that could explain the absence of transitional B-cells in peripheral blood (**Figure 5**). Of patients 1 to 5, who all lacked transitional B-cells in peripheral blood, patients 4 and 5 had a normal precursor B-cell development in bone marrow. Patients 6 and 7 had normal numbers of transitional B-cells in blood, but in patient 7 no pro B and pre B I cells could be detected, which was also seen in patient 1 (**Figure 5**).

In bone marrow samples, mature B-cells (i.e., non-precursor B-cells) are present mainly because of peripheral blood contamination. The percentage of these so-called “bone marrow mature B-cells” in AA was comparable to the healthy donor samples. Within this bone marrow mature B-population, the transitional B-cells were also relatively reduced in AA compared to the donors (**Supplementary Figure S7**). The combined data show that transitional B-cells are strongly reduced in AA, despite complete bone marrow precursor B-cell development until the immature B-cell stage. In addition, about half of the AA patients had a remarkable reduction in pro-B cells and pre-B-I cells.

### 3.7 A shift towards more precursor B-cells and less CD34^+^ progenitors in a subgroup of AA patients

**Figure 6:**
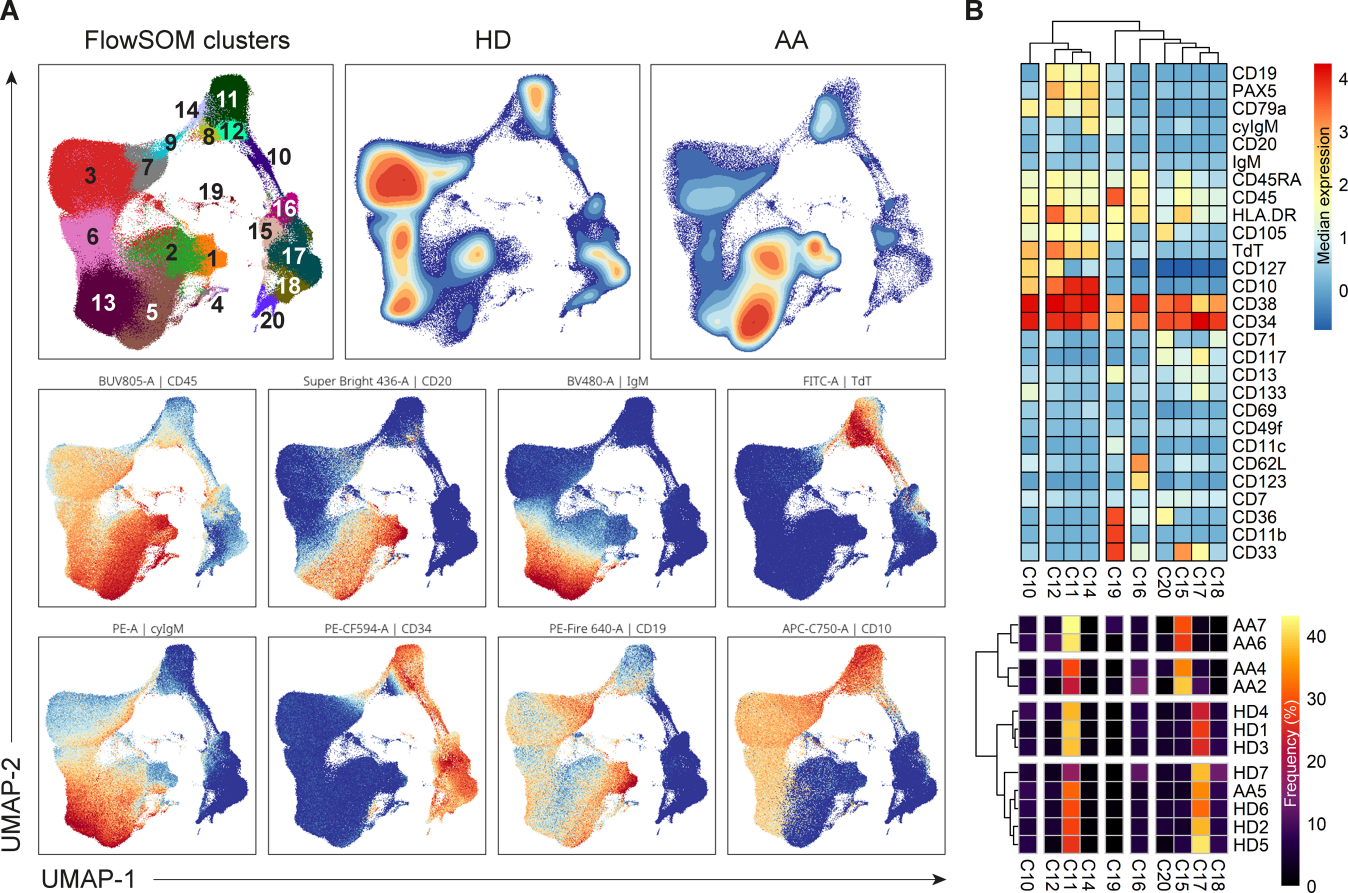
Analysis of CD34^+^ progenitors and precursor B-cells. **(A)** UMAP and FlowSom clustering of CD34^+^ progenitors and precursor B-cells, based on all fluorochrome conjugated markers except the live/dead marker, CD10, CD15, CD38 and IgD. An equal number of cells per group (HD vs AA) was included in the analysis, resulting in a minimum of 2.168 and a maximum of 52.376 events per sample. **(B)** A heatmap based on median expression levels and proportions of each individual CD34^+^ cluster. Patients 1 and 3 were excluded due to an insufficient number of events.

Dimensionality reduction (UMAP) and FlowSOM clustering were performed on the CD34^+^ progenitors and precursor B-cells (**Figure 6A**; **Supplementary Figure S8**). In total, 20 clusters were identified, each representing a unique phenotype (**Table 3**). The clusters on the lower right (cluster 15-20) represented the CD34^+^ progenitors and clusters on the left part (cluster 1-3 and 4-14) of the UMAP the precursor B-cells which were positive for CD19, CD10 and had a gradual increase in the expression of cyIgM. In patient 1, 2, 3 and 7, the distribution in percentages of CD34^+^ progenitors and precursor B-cells was shifted towards more CD34^-^ precursor B-cells and less CD34^+^ cells as compared to the healthy donors (**Figure 6A**; **Supplementary Table S5**).

**Table 3:** Definitions of CD34^+^ progenitors and precursor B-cell clusters.

		Phenotype	Cluster
CD34+ Progenitors		EMP	20
		EMP	19
		EMP and some MPP	18
		MPP and GMP+LMPP, some pro B	17
		Majority late GMP and some GMP+LMPP and EMP	15
		Late GMP and some pro B	16
	Precursor B-cells	Pro B	10
		Pre B I and some pre B II	12
		Pre B I	11
		Pre B I	14
		Pre B I and pre B II	8
		Pre B II	9
		Pre B II	7
		Pre B II	3
		Pre B II and some immature B	2
		Pre B II and some immature B	1
		*Artefact*	*4*
		Immature B	6
		Immature B	13
		Immature B	5

GMP, granulocyte-macrophage progenitor; MPP, multipotent progenito; LMPP, lymphoid-primed multipotent progenitor; EMP, erythro-myeloid progenitor.

In depth analysis of progenitor cells was performed by selecting only the CD34^+^progenitor cells (**Figure 6B**). Patients 1 and 3 were excluded from analysis due to an insufficient number of events. Interestingly, patient 5 clustered with the healthy donors, indicating no aberrant distribution in CD34^+^ progenitor cells. For all other AA patients, differences in distribution could be observed compared to healthy donors. Cluster 15, characterized mostly by late granulocyte-macrophage progenitors (GMPs) and some GMP + lymphoid-primed multipotent progenitors (LMPPs) and erythro-myeloid progenitors (EMPs), represented 27.5 to 39% of CD34^+^ cells in these patients, as compared to a mean of 5.6% in healthy donors. Only 2.7 to 9.6% of these patients’ CD34^+^ cells were represented by cluster 17, defined as multipotent progenitors (MPPs), GMP+LMPP and some pro B, as compared to a mean of 32.2% in healthy donors. Cluster 18, characterized by EMPs and some MPPs, was also reduced in this subgroup of patients, with a proportion of 0 to 1.3%, compared to a mean of 7.9% in healthy donors. Together, these results indicate a shift from CD34^+^ progenitors towards precursor B-cells in AA patients compared to healthy donors.

## 4 Discussion

Although the widely accepted hypothesis states that AA is caused by immune dysregulation or auto-immunity, the exact mechanism is still not fully understood. As part of the current standard diagnostic pathway for pediatric patients with BMF, we frequently observed lower peripheral NK- and B-cell counts in pediatric AA patients. Interestingly, this observation was not only true in comparison to healthy controls but also when compared to pediatric patients with constitutional hypoplastic BMF such as in telomere biology disorders (TBD), NK- and B-cell counts were frequently decreased in peripheral blood (3). Therefore, in this study, we aimed to investigate the presence and characteristics of NK- and B-cells in peripheral blood and bone marrow of pediatric AA patients in comparison to age-matched healthy bone marrow donors.

We categorized pediatric patients as AA if patients were healthy until the onset of BMF, unexposed to cytotoxic drugs or radiation and if other known causes of BMF, mainly inherited bone marrow failure syndromes (IBMFS) and (pre)malignant conditions, were excluded. In parallel to adult patients with acquired/immune AA, this category of pediatric patients might suffer from an auto-immune driven process affecting healthy hematopoiesis. In immune AA, the most hypothesized disease mechanism includes a viral infection driven T-cell attack towards the hematopoietic stem cell, although the initiating antigenic target for this immune response remains elusive. In addition, ineffective inhibition of the T-cell response by regulatory cells and cytokines maintaining immune dysregulation, auto-immunity and micro-environmental marrow inflammation have been proposed to facilitate the disruption of hematopoiesis in these patients (24).

In this study, we focused on a potential role for NK- and B-cells in pathogenesis. We observed a reduced absolute number of NK-cells in peripheral blood of all patients. However, the considered immunoregulatory CD56^bright^ NK-cell subset (25, 26) was enriched in three patients (1, 6 and 7) and even normal absolute CD56^bright^ counts were detected in two of them. After HSCT, which is like AA also an inflammatory setting, absence of T-cells is associated with expansion of CD56^bright^ NK-cells (27). Therefore, the expansion of the CD56^bright^ NK-cells might be a result of the low T-cell numbers, as observed in patient 1 and 6. The loss of CD45RA on this subset suggests that these NK-cells are activated. Accordingly, in the blood of both multiple myeloma patients and HSCT recipients a similar loss of CD45RA was observed (28). However, in spite of normal degranulation, IFN-γ production and perforin expression were reduced in this specific patient subgroup, suggesting the function of NK-cells to be impaired. Interestingly, the observed reduction in NK-cell function was not limited to the CD56^bright^ NK-cells. Impaired NK-cell activity has been reported in multiple auto-immune diseases, including multiple sclerosis (MS) (29), systemic lupus (SLE) (30), rheumatoid arthritis (RA) (31) and adult AA (12, 32, 33). Overall, these results imply that diminished NK-cell function in a subgroup of AA patients might underly the auto-immunity. Importantly, degranulation, IFN-γ production and intracellular perforin of AA patients with reduced CD56^bright^ NK-cell counts (patient 2, 3, 4 and 5) were within the range of healthy controls. Of note, NK-cells were found to be relatively increased in the bone marrow. Additional analyses are required to pinpoint the exact role and cellular interactions of NK-cells in pediatric AA.

In our pediatric AA cohort, the absolute numbers of B-cells were reduced in peripheral blood, which concerned all B-cell subsets. Although a greater sample size is required to confirm the observation, the trend towards reduction or even absence of transitional B-cells was remarkable and in line with a previous study in adult AA (13). Transitional B-cells are regarded as the intermediates between bone marrow precursor-B and peripheral naive mature B-cells. However, this population is also suggested to contain B-regulatory cells (Bregs), which have specific B-cell receptor repertoire characteristics outside the trajectory of gene loss or gain between precursor B - naive mature stages (34). Reduction of these Breg cells in AA was previously hypothesized to be involved in defective inhibition of auto-reactive T cells (13). Interestingly, even in AA patients with normal distribution of precursor B-cell stages in the bone marrow, the transitional compartment was reduced, suggesting a partial differentiation failure from immature to transitional B cells or selective loss, as naive B-cells were not reduced. It might be that the specific reduction of Bregs contributes to the development of AA. However, in depth functional investigations on the normal role of Bregs are necessary to determine whether this phenomenon indeed contributes to the pathogenesis in AA and if Bregs can be used as diagnostic or prognostic markers in immune AA.

Furthermore, in depth analysis of the bone marrow CD34^+^ progenitors and precursor B-cells revealed a shift from CD34^+^ progenitors towards more precursor B-cells in a subgroup of AA patients as compared to the healthy donors. Reduced CD34+ progenitor cells are a hallmark of AA, but it is remarkable that the B-cell lineage remains relatively unaffected in comparison to the myeloid lineages, which are completely absent or destroyed. This might be partly explained by a difference in cellular turnover between the lineages. Further research is necessary to better understand this observation.

Although patients in this study were selected after exclusion of constitutional BMF syndromes to increase the probability of an underlying immune based disease mechanism, we were intrigued by the identification of a germline VUS in 3 out of 7 patients in BMF related genes (*SAMD9, SAMD9L* and *RPS24*) and a pathogenic *BRCA1* mutation in another patient. Despite the lack of evidence for the pathogenicity of these variants, especially the *SAMD9* and *RPS24* mutations might suggest a predisposing role for the development of AA. Comparable observations were reported for *TERC, TERT*, and *SLX4* variants of unknown significance predisposing or causing AA (35). Hypothetically, these variants might not be pathogenic as a single factor causing BMF as compared to other known pathogenic *SAMD9* and *RPS24* mutations in IBMFS but might render patients more susceptible to BMF within a multifactorial disease mechanism. Functional studies are essential to provide additional information on whether these variants predispose patients to AA by intrinsic increased HSC vulnerability to inflammation, increased T-cell auto-reactivity directly or via accumulation of additional somatic mutations and/or reduced inhibitory potential of the regulatory immune cells (24).

Limitations of this study include low patient numbers and low numbers of bone marrow cells to study and quantify findings due to hypocellularity of the bone marrow.

Pediatric immune AA patients may be successfully treated by IST to avoid risks of HSCT. However, diagnostic markers to identify immune AA and prognostic markers to predict IST response are required to personalize treatment. Therefore, high-dimensional single-cell studies in larger homogeneous patient cohorts are crucial for the identification of disease-causing events that could serve as targets for therapy.

## Data Availability

The raw data supporting the conclusions of this article will be made available by the authors, without undue reservation.
