# Supplementary material for: Potential role of B- and NK-cells in the pathogenesis of pediatric aplastic anemia through deep phenotyping
Source: Front Immunol. 2024 Aug 20;15:1328175. doi: 10.3389/fimmu.2024.1328175 (PMC11368747; doi:10.3389/fimmu.2024.1328175)
Supplement: Supplementary file 1 [file DataSheet1.pdf]

Supplementary Material

Supplementary Table 1. Antibodies spectral flow cytometry. BMMCs were stained with 38 fluorochrome-conjugated antibodies (BM). A two-tube flow cytometry panel was designed to study the lymphocytes in the peripheral blood (TNK and TB). Panel to study function of peripheral blood NK cells (NK function).

CD code	Target	Surface / intracellular	Manufacturer	Clone	Fluorochrome	BM	TNK	TB	NK function
CD3		surface	Biolegend	UCHT1	BV510	x			
CD3		surface	Biolegend	UCHT1	BV785		x		
CD3		surface	Biolegend	UCHT1	AF594			x	
CD3		surface	Biolegend	UCHT1	BV421				x
CD4		surface	eBioscience	SK3	AF532		x		
CD4		surface	Biolegend	SK3	PE-Fire640			x	
CD7		surface	eBioscience	eBio124-1D1	AF532	x		x	
CD7		surface	BD Biosciences	M-T701	R718		x		x
CD7		surface	Beckman Coulter	3A1E-12H7	RD1				
CD8		surface	BD Biosciences	RPA-T8	BUV395		x	x	
CD10		surface	Cytognos	HI10a	APC-C750	x			
CD11b		surface	Biolegend	MI/70	BV750	x	x		
CD11c		surface	BD Biosciences	B-Ly6	BUV661	x			
CD13		surface	BD Biosciences	WM15	BV605	x			
CD14		surface	Invitrogen	TuK4	Qdot 800	x	x		
CD15		surface	Biolegend	W6D3	PerCP	x			
CD16		surface	BD Biosciences	3G8	BUV615	x	x		
CD16		surface	Biolegend	3G8	AF647			x	
CD16		surface	Biolegend	3G8	BV480				x
CD19		surface	Biolegend	HIB19	PE-Fire640	x			
CD19		surface	BD Biosciences	HIB19	BV786				x
CD19		surface	BD Biosciences	SJ25C1	BV786			x	
CD20		surface	eBioscience	2H7	SB436	x			
CD21		surface	BD Biosciences	B-ly4	APC			x	
CD24		surface	BD Biosciences	ML5	BV650			x	
CD25		surface	BD Biosciences	2A3	BUV615			x	
CD27		surface	Biolegend	QA17A18	APC-Fire810		x	x	
CD28		surface	BD Biosciences	CD28.2	BUV661		x	x	
CD31		surface	BD Biosciences	WM59	BV750			x	
CD33		surface	Biolegend	WM53	PE-Cy5	x			

CD34		surface	BD Biosciences	581	PE-CF594	x			
CD36		surface	Beckman Coulter	FA6.152	PacB	x			
CD38		surface	BD Biosciences	HIT2	PerCP-Cy5.5		x		
CD38		surface	Biolegend	HIT2	BV605			x	
CD38		surface	Biolegend	S17015F	PE-Fire810	x			
CD45		surface	BD Biosciences	HI30	BUV805	x	x	x	
CD45		surface	BD Biosciences	2D1	APC-H7				x
CD45RA		surface	Biolegend	HI100	BV570	x	x	x	
CD45RA		surface	Biolegend	MEM-56	PE-TxR				x
CD45RO		surface	BD Biosciences	UCHL1	BUV496			x	
CD49f		surface	eBioscience	GoH3	PerCP-ef710	x			
CD56		surface	Beckman Coulter	N901 (NKH-1)	APC	x			
CD56		surface	BD Biosciences	NCAM16.2	BV650		x		x
CD56		surface	BD Biosciences	NCAM16.2	BUV737			x	
CD57		surface	Biolegend	HNK-1	PacB		x		x
CD62L		surface	Biolegend	DREG-56	BV650	x			
CD69		surface	BD Biosciences	FN50	BUV737	x	x		
CD69		surface	BD Biosciences	FN50	BV785				x
CD71		surface	BD Biosciences	M-A712	BV786	x			
CD74		surface	BD Biosciences	LN2	BV711			x	
CD79a		intracellular	Biolegend	HM47	PE-Cy7	x			
CD90		surface	BD Biosciences	5E10	BUV563	x			
CD95	FAS	surface	Biolegend	DX2	PE-Dazzle594			x	
CD103		surface	Biolegend	Ber-ACT8	PE-Fire640		x		
CD105		surface	Miltenyi biotec	REA794	VioBright 515	x			
CD117		surface	Biolegend	104D2	BV711	x	x		
CD123		surface	Biolegend	6H6	APC-Fire810	x			
CD127		surface	eBioscience	eBioRDR5	PE-Cy5.5	x	x	x	
CD133		surface	BD Biosciences	293C3	BV421	x			
CD159a	NKG2A	surface	Beckman Coulter	Z199	PE-Cy7		x		x
CD159c	NKG2C	surface	BD Biosciences	134591	BV421		x		
CD159c	NKG2C	surface	BD Biosciences	134591	RB744				x
CD160		surface	BD Biosciences	BY55	AF488		x		
CD183	CXCR3	surface	BD Biosciences	1C6/CXCR3	BV421			x	
CD185	CXCR5	surface	Miltenyi biotec	REA103	PE-Vio770			x	
CD186	CXCR6	surface	Biolegend	K041E5	PE-Dazzle594		x		
CD194	CCR4	surface	Biolegend	L291H4	PE-Fire810			x	
CD195	CCR5	surface	BD Biosciences	2D7	RY586		x		
CD196	CCR6	surface	BD Biosciences	11A9	R718			x	
CD197	CCR7	surface	Biolegend	G043H7	APC-Fire750		x	x	

CD226	DNAM1	surface	BD Biosciences	DX11	BUV563		x		
CD235a		surface	BD Biosciences	GA-R2(HIR2)	BUV395	x			
CD244	2B4	surface	Biolegend	C1.7	BV605		x		
CD279	PD-1	surface	BD Biosciences	EH12.1	RB780		x		
CD366	TIM-3	surface	R&D Systems	344823	PE		x		
	CX3CR1	surface	Miltenyi biotec	REA385	APC		x		
	FVDUV455	intracellular	eBioscience		eFluor455UV	x			
	GranzymeB	intracellular	Biolegend	QA16A02	AF700				
	HLA-DR	surface	BD Biosciences	G46-6	BUV496	x	x		
	IFN-γ	intracellular	BD Biosciences	4S.B3	RB780				x
	IgA	surface	Miltenyi biotec	IS11-8E10	FITC			x	
	IgA	surface	Miltenyi biotec	IS11-8E10	PE			x	
	IgD	surface	Biolegend	IA6-2	PerCP-Cy5.5	x		x	
	IgE	surface	eBioscience	IgE21	FITC			x	
	IgG	surface	BD Biosciences	G18-145	PE			x	
	IgM	surface	BD Biosciences	G20-127	BV480	x		x	
	IgM	intracellular	DAKO	polyclonal	PE	x			
	Ki-67	intracellular	Miltenyi Biotec	REA183	vioR720	x			
	KLRG1	surface	eBioscience	13F12F2	PerCP-eF710		x		
	PAX5	intracellular	BD Biosciences	1H9	AF647	x			
	perforin	intracellular	eBioscience	dG9	PerCP-ef710				x
	TCRgd	surface	BD Biosciences	11F2	BV510		x	x	
	TCRVa7.2	surface	BD Biosciences	OF-5A12	BV480		x		
	TdT	intracellular	DAKO	HT-6	FITC	x			
	TIGIT	surface	eBioscience	MBSA43	AF647		x		x

Supplementary Table 2. Characteristics of age-matched healthy donors.

Donor	Gender	Age at sampling (y)	CMV status
**HD1**	f	8	Neg
**HD2**	m	12	Pos
**HD3**	m	6	Neg
**HD4**	m	2	Neg
**HD5**	m	9	Pos
**HD6**	m	10	Pos
**HD7**	m	6	Pos

Supplementary Table 3. Absolute lymphocyte counts (cell count per μL) within peripheral blood. Values were compared to age-matched reference values (1, 2). Low values were indicated in red. Grey indicated the absence of age-matched reference values.

	Age at sampling (y)	T-cells	NK-cells	NK^{dim}	NK^{bright}	B-cells	Transitional	Naïve mature	MZ/ Natural effector	IgM memory	Switched memory IgA^{+}	Switched memory IgG^{+}	DN memory IgM^{+}	DN memory IgA^{+}	DN memory IgG^{+}	Plasmablast
D1	8.3	1803	235	216	20	301	29	156	8	6	13	32	5	5.7	35	1.8
D2	12.1	1505	364	343	20	400	67	237	24	15	13	25	5	1.2	7	1.3
D3	5.9	1058	187	160	27	469	97	281	15	15	7	13	14	3.0	12	2.7
D4	1.7	1956	188	168	21	962	320	567	11	10	4	12	17	0.9	9	5.5
D5	9.4	2536	194	180	14	542	71	365	16	11	8	25	13	2.9	18	0.4
D6	10.2	2083	251	231	20	421	56	243	13	22	9	18	29	5.7	17	1.1
D7	6.5	3359	417	395	22	683	116	429	25	21	11	18	26	4.2	19	3.7
1	15.9	676	12	9	4	104	>1	77	6	6	3	2	7	0.6	1	0.1
2	8.7	758	89	83	7	88	>1	53	5	5	4	10	3	0.7	5	0.4
3	2.7	4165	119	112	6	414	2	375	9	3	1	5	6	0.7	5	0.5
4	18.2	768	37	34	4	74	>1	36	2	5	4	2	15	2.9	3	0.8
5	5.1	1180	17	15	2	76	1	69	1	>1	>1	1	>1	0.0	1	0.3
6	17.1	345	41	26	16	112	16	86	2	>1	>1	>1	3	0.2	1	0.1
7	3.3	2597	87	40	47	643	88	366	61	30	13	32	19	4.1	23	1.0

Supplementary Table 4. Estimation of blood contamination of bone marrow samples. Blood contamination was estimated by adding the frequency of $CD69^-$ T-, $CD69^-$ NK- and mature B-cells.

Donor/patient	% Blood contamination
D1	25
D2	39
D3	30
D4	32
D5	50
D6	28
D7	50
AA1	88
AA2	67
AA3	77
AA4	47
AA5	72
AA6	37
AA7	75

Supplementary Table 5. Distribution of $CD34^+$ progenitors and precursor B-cells per patient.

	% $CD34^+$ progenitor cells	% $CD34^+$ precursor B-cells	% $CD34^-$ precursor B-cells
HD1	11.5	10.3	78.2
HD2	14.2	9.4	76.4
HD3	9.6	10.2	80.2
HD4	7.3	8.9	83.7
HD5	25.1	14.2	60.8
HD6	14.8	11.0	74.1
HD7	15.8	5.9	78.3
AA1	0.2	0.0	99.8
AA2	2.7	1.3	96.0
AA3	1.6	3.4	95.0
AA4	15.6	12.1	72.4
AA5	10.5	9.2	80.3
AA6	10.4	15.8	73.8
AA7	0.2	0.2	99.6

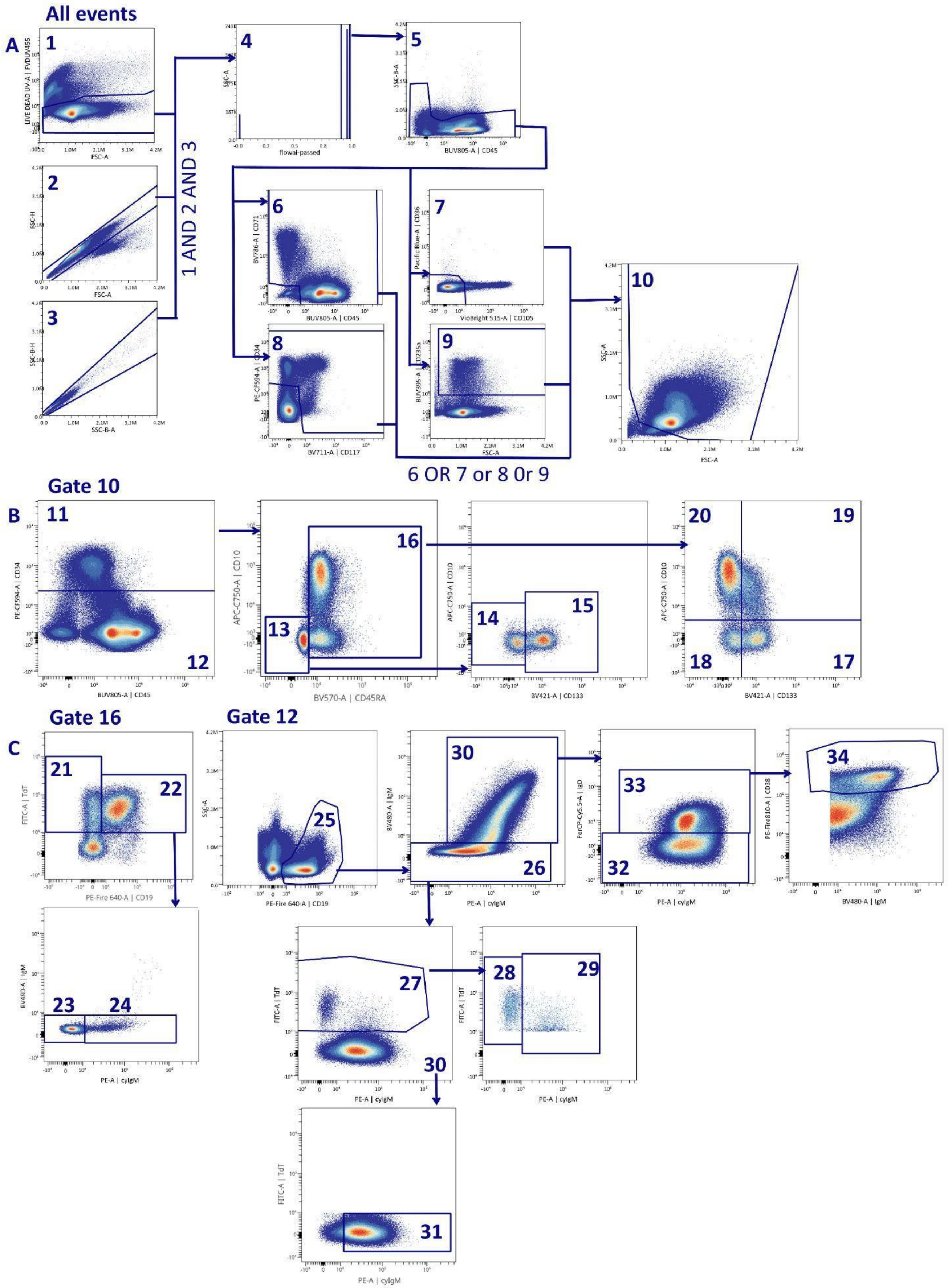
A
All events
1
2
3
1 AND 2 AND 3
4
5
6
7
8
9
10
6 OR 7 or 8 0r 9
LIVE DEAD UV-A | FVDUV455
FSC-A
SSC-H
SSC-B-H
SSC-B-A
SSC-A
flowai-passed
BUV805-A | CD45
BV786-A | CD71
Pacific Blue-A | CD36
VioBright 515-A | CD105
PE-CF594-A | CD34
BV711-A | CD117
BUV395-A | CD235a
B
Gate 10
11
12
13
14
15
16
17
18
19
20
APC-C750-A | CD10
BV570-A | CD45RA
BV421-A | CD133
C
Gate 16
Gate 12
21
22
23
24
25
26
27
28
29
30
31
32
33
34
FITC-A | TdT
PE-Fire 640-A | CD19
BV480-A | IgM
PE-A | cyIgM
PerCP-Cy5.5-A | IgD
PE-Fire810-A | CD38

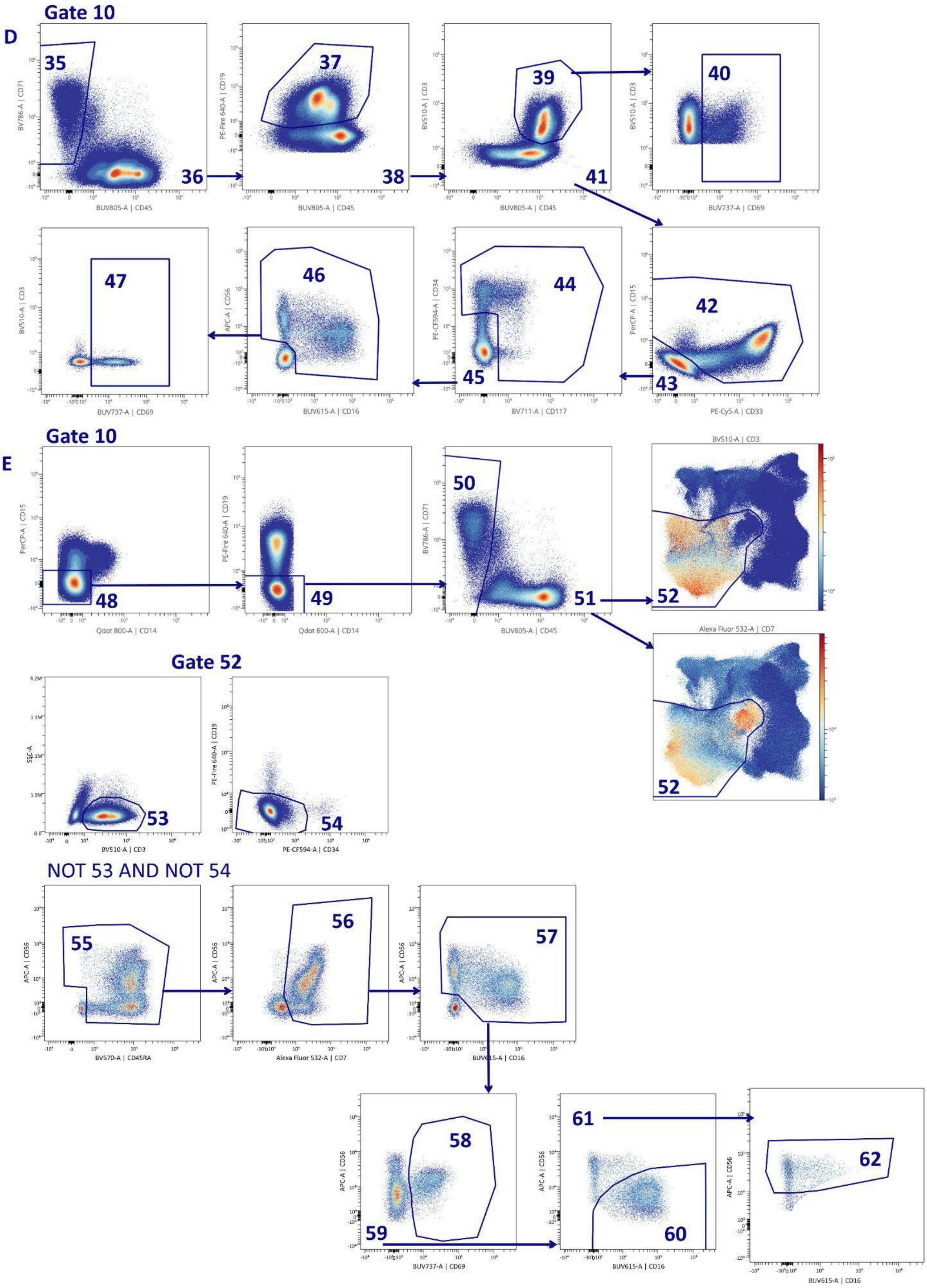

Gate 10
D
35
36
37
38
39
40
41
42
43
44
45
46
47
BV786-A | CD71
BUV805-A | CD45
PE-Fire 640-A | CD19
BV510-A | CD3
BUV737-A | CD69
PerCP-A | CD15
PE-Cy5-A | CD33
PE-CF594-A | CD34
BV711-A | CD117
APC-A | CD56
BUV615-A | CD16
Gate 10
E
48
49
50
51
52
Qdot 800-A | CD14
Alexa Fluor 532-A | CD7
Gate 52
53
54
SSC-A
NOT 53 AND NOT 54
55
56
57
58
59
60
61
62
BV570-A | CD45RA

Supplementary Figure 1. Gating strategy for BM mononuclear cells. **A)** (1-3) viable singlets; (4) FlowAI passed events; (5) SSC low cells; (6-9) CD71^{+} or CD45^{+} or CD36^{+} or CD105^{+} or CD34^{+} or CD117^{+} or CD235a^{+} events; (10) mononuclear cells. **B)** Gating strategy for (11) CD34^{+} hematopoietic progenitor cells (HPC); (13) CD45RA^{-} cells; (14) CD34^{+}CD45RA^{-}CD133^{-} erythro-myeloid progenitor (EMP); (15) CD34^{+}CD45RA^{+}CD133^{+} multipotent progenitor (MPP); (16) CD45RA^{+} cells; (17) CD45RA^{+}CD133^{+}CD10^{-} granulocyte-macrophage progenitor (GMP) or lymphoid-primed multipotent progenitor (LMPP); (18) CD45RA^{+}CD133^{-}CD10^{-} late GMP; (19) multi lymphoid progenitor (MLP) CD45RA^{+}CD133^{+}CD10^{+} and (20) CD45RA^{+}CD133^{-}CD10^{+} B cell biased lymphoid progenitor (BLP)**. C)** Gating strategy for CD34^{+} BM precursor B-cells; (21) CD19^{-}TdT^{+} pro B; (23) cyIgM^{-} pre B I; (24) cyIgM^{+} pre B II. (25) CD34^{-}CD19^{+} B-cells; (26) sIgM^{-} B-cells; (27) TdT^{+} B-cells; (28) TdT^{+}cyIgM^{-} pre B I; (29,31) cyIgM^{+} pre B II and (32) sIgM^{+}sIgD^{-} immature B-cells; (33) sIgM^{+}sIgD^{+} B-cells and (34) CD38^{++} transitional B-cells. **D)** Gating strategy for BMMC composition; (35) CD45^{-}CD71^{+} erythroid cells; (37) CD19^{+} B-cells; (39) CD3^{+} T-cells; (40) CD3^{+}CD69^{+} Trm-cells; (42) CD33^{+}CD15^{+-} myeloid cells; (44) CD34^{+-}CD117^{+-}Progenitor cells; (46) CD56^{+}CD16^{+-}NK-cells and (47) CD56^{+}CD69^{+} ltNK-cells. **E)** Gating strategy for NK-cell populations; (48) CD14^{-}CD15^{-} non myeloid cells; (49) CD19^{-} non B-cells; (51) CD71^{-} non erythroid cells; (52) EmbedSOM gate T- or NK-cells; (53-54) CD3^{-}CD19^{-}CD34^{-} cells; (55) CD56^{+}CD45RA^{+-} cells; (56) CD56^{+}CD7^{+} NK-cells; (57) CD56^{+}CD16^{+-} NK-cells; (58) CD69^{+} ltNK-cells; CD56^{dim} NK-cells and (62) CD56^{bright} NK-cells.

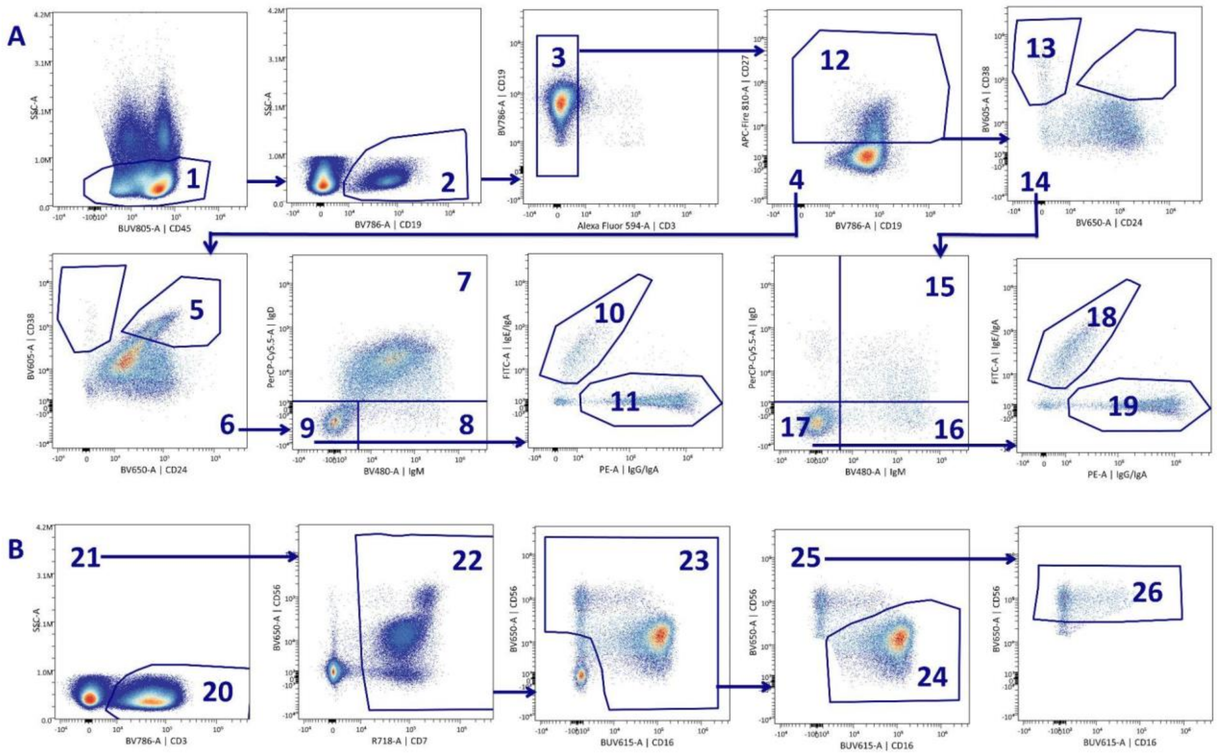

Supplementary Figure 2. Gating strategy for peripheral blood B-cells after cleanup gating. **A)** (1) $CD45^{+}SSC^{low}$ lymphocytes were gated (2-3) $CD19^{+}CD3^{-}$ B-cells; (4) $CD27^{-}$ B-cells; (5) $CD24^{++}CD38^{++}$ transitional B-cells; (6) $CD27^{-}CD38^{dim}$ B cells; (7) $sIgM^{+}sIgD^{+}$ naïve mature B-cells; (8) $CD27^{-}sIgD^{-}$ DN memory $sIgM^{+}$ B-cells; (9) DN memory $sIgM^{-}$ B-cells; (10) $sIgA^{+}$ DN memory B-cells; (11) $sIgG^{+}$ DN memory B-cells; (12) $CD27^{+}$ memory B-cells; (13) plasmablasts; (14) $CD27^{+}CD38^{dim}$ B-cells; (15) MZ/natural effector B-cells; (16) $sIgM^{+}$ memory B-cells; (17) $sIgD^{-}sIgM^{-}$ switched memory B-cells; (18) $sIgA^{+}$ switched memory B-cells and (19) $sIgG^{+}$ switched memory B-cells. **B)** Gating strategy for peripheral blood NK-cells after cleanup gating and gating on lymphocytes: (20) $CD3^{+}$ T-cells; (21) $CD3^{-}$ lymphocytes; (22) $CD7^{+}CD56^{+}$ NK-cells; (23) $CD56^{+}CD16^{+-}$ NK-cells; (24) $CD56^{dim}$ NK-cells; (25) NK-cells$^{not\ dim}$ and (26) $CD56^{bright}$ NK-cells.

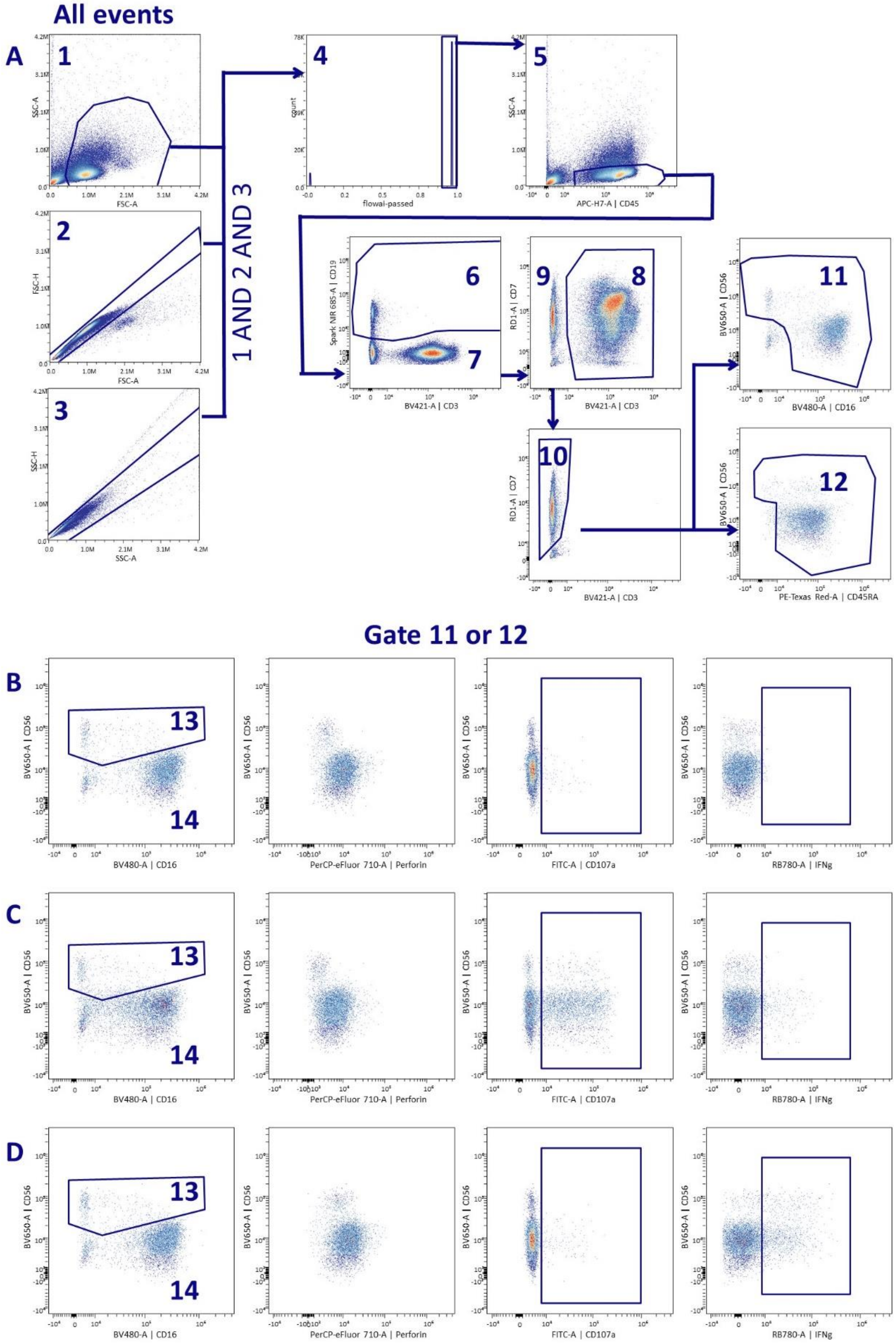

Supplementary Figure 3. Gating strategy NK-cells after incubation of PBMC for 4 hrs. to study NK-cell function. A,B) PBMC incubated in medium. (1-3) Gating single cells; (4) FlowAI passed events; (5) CD45$^+$SSC^{low} lymphocytes; (6) CD19$^+$ B-cells; (7) CD19$^-$ cells; (8) CD3$^+$ T-cells; (9)

$CD19^-CD3^-$ lymphocytes; (10) $CD7^+$ cells; (11) $CD56^+CD16^{+-}$ NK-cells and (12) $CD56^+CD45RA^+$ NK-cells; (13) $CD56^{bright}$ NK-cells and (14) $CD56^{dim}$ NK-cells. C) PBMC incubated with K562 target cells (1:1 effector:target ratio). (D) PBMC incubated in medium with 10 ng/mL IL-12, 10 ng/mL IL-15 and 20 ng/mL IL-18.

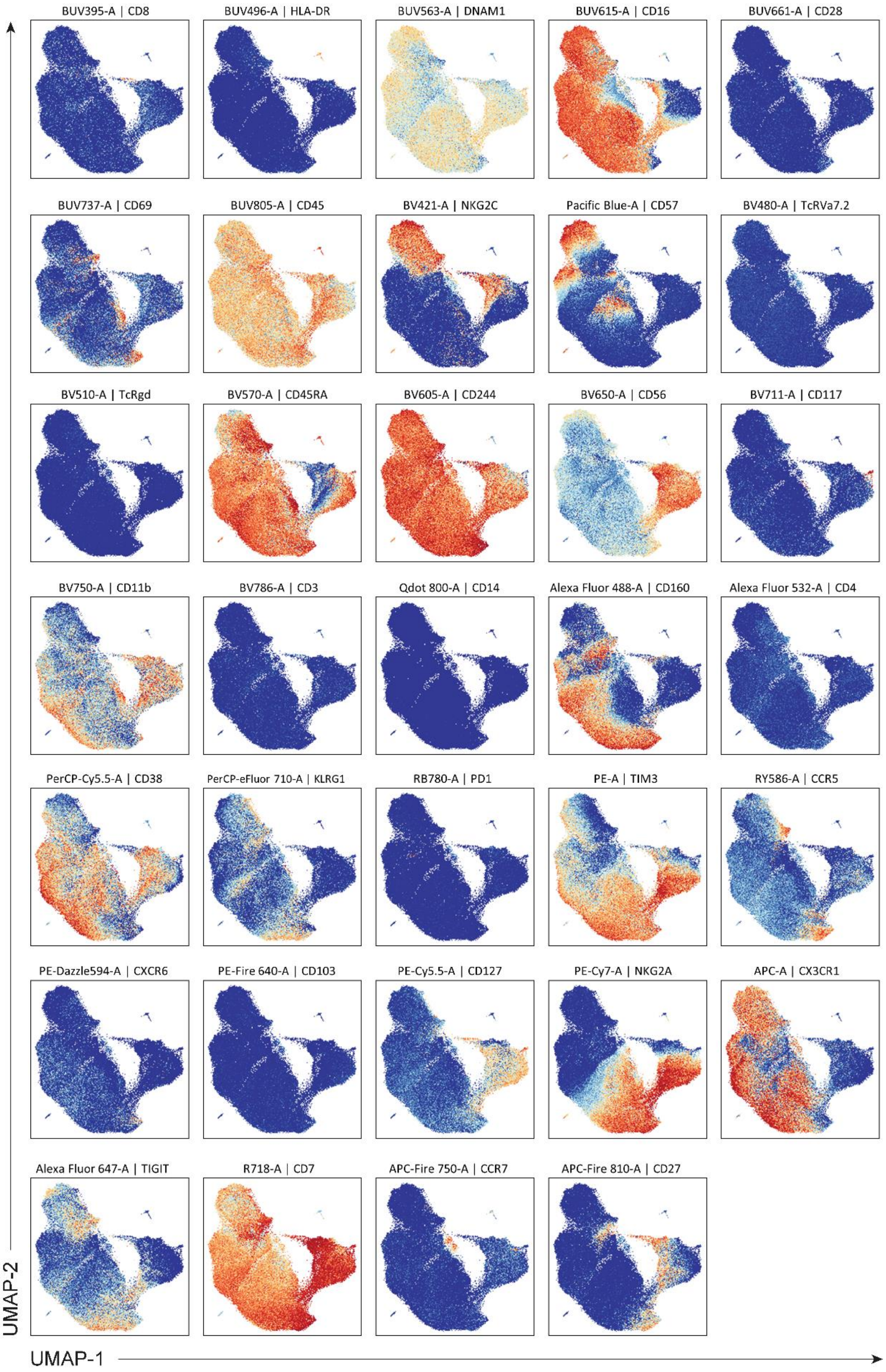

Supplementary Figure 4. UMAP of peripheral blood NK-cells. UMAP was based on all fluorochrome conjugated markers in the panel, except the live/dead marker. An equal number of cells per group (HD vs AA) was included.

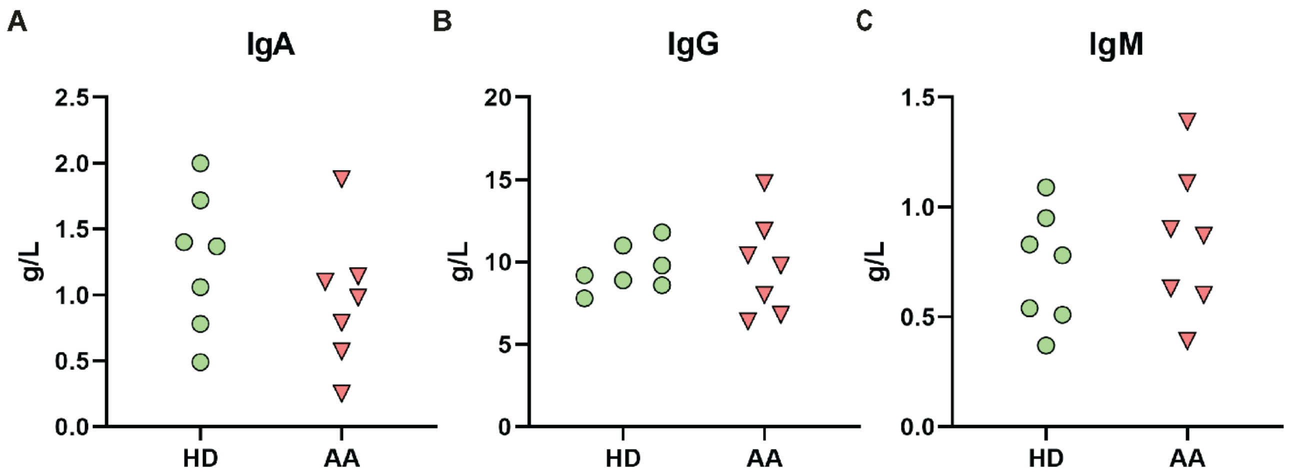

Supplementary Figure 5. Ig values in the peripheral blood. Amount of **A)** IgA, **B)** IgG and **C)** IgM in the peripheral blood of AA patients compared to age-matched healthy donors (HD). Data were compared using the Mann-Whitney U test. After multiple testing correction using the false discovery rate (FDR) no significant differences were observed.

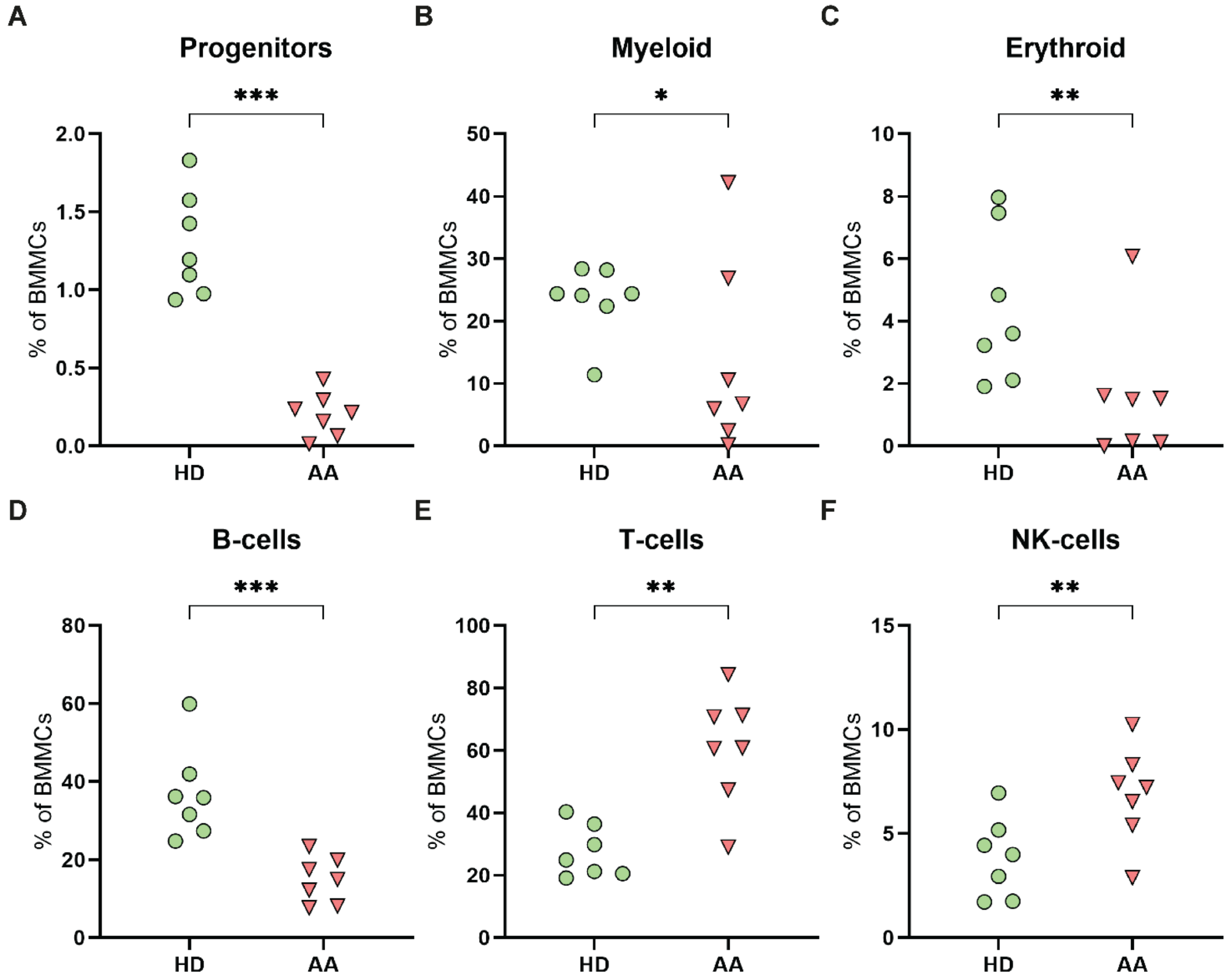

Supplementary Figure 6. Bone marrow composition. The proportion of **A)** progenitor, **B)** myeloid, **C)** erythroid, **D)** B-cells, **E)** T-cells and **F)** NK-cells in the bone marrow of AA patients compared to healthy donors (HD). Data were compared using the Mann-Whitney U test. Significant values after multiple testing correction using the false discovery rate (FDR) are indicated: ***, P<0.001; **, P<0.01, *, P<0.05.

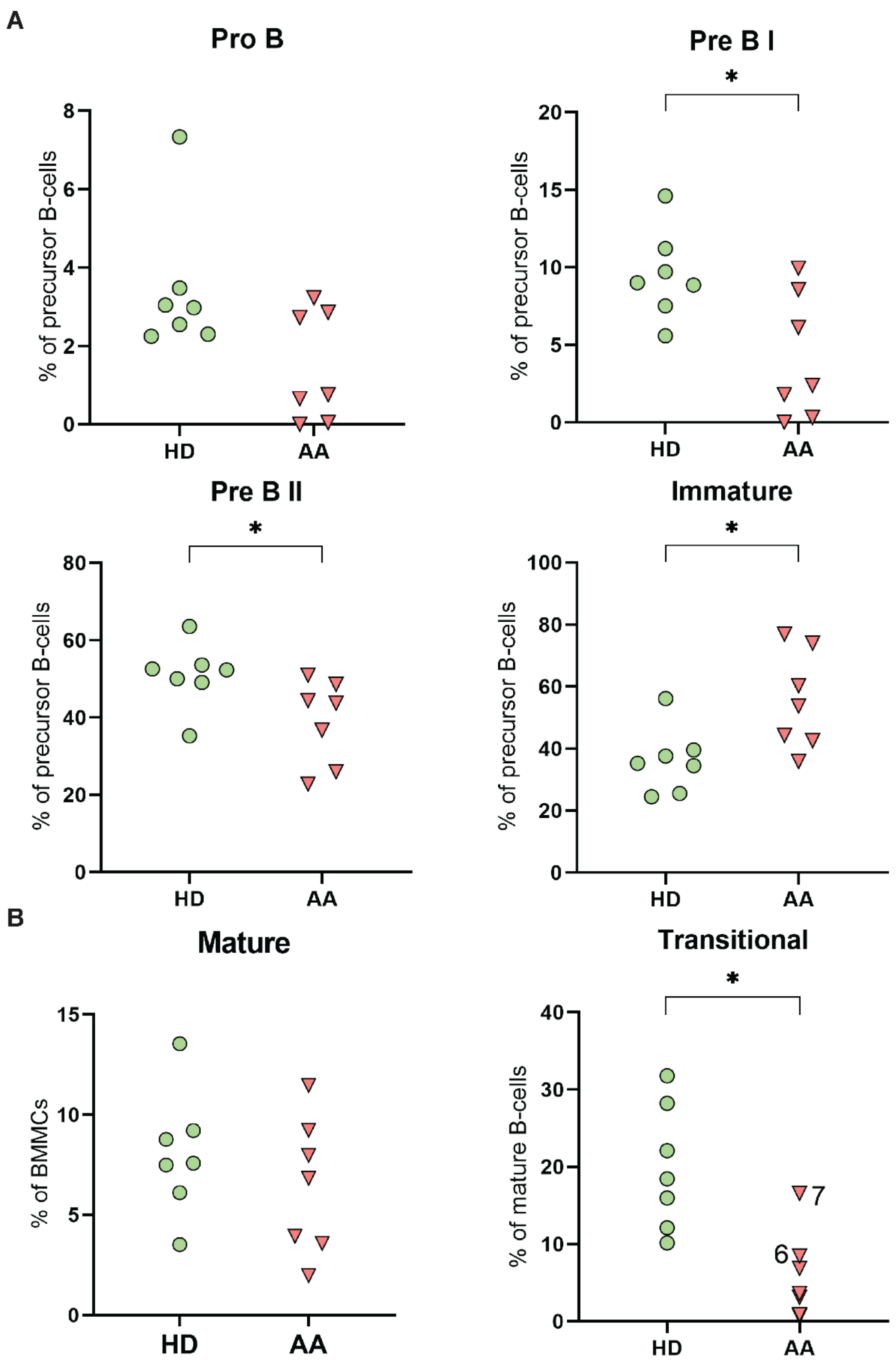

Supplementary Figure 7. Relative composition of the B-cell compartment in the bone marrow samples. **A)** Percentage of bone marrow derived precursor B-cells in AA and healthy donors (HD). **B)** Bone marrow mature B-cells. Data were compared using the Mann-Whitney U test. Significant values after multiple testing correction using the false discovery rate (FDR) are indicated: *, P<0.05.

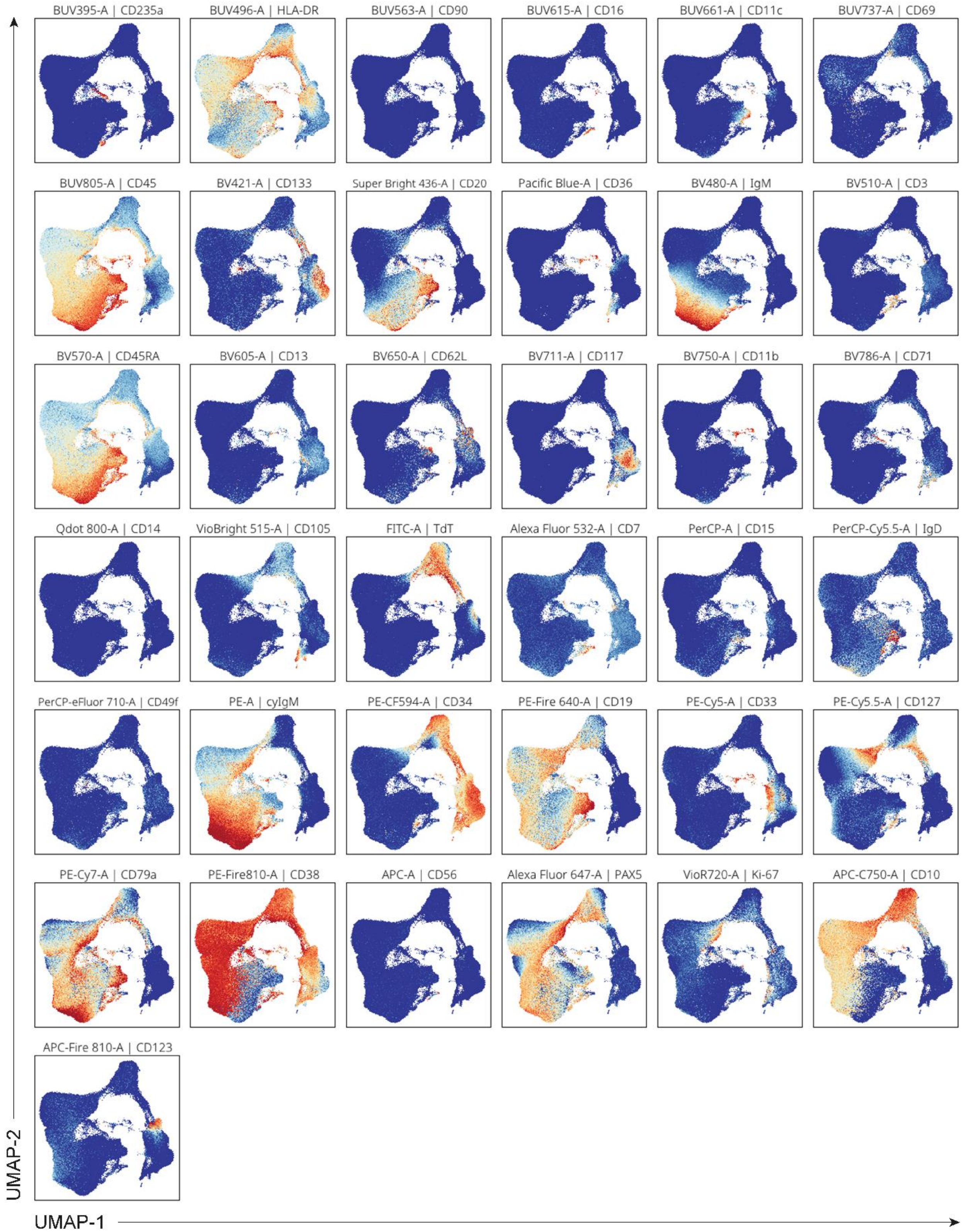

Supplementary Figure 8. UMAP of CD34$^+$ progenitors and precursor B-cells. UMAP was based on all fluorochrome conjugated markers in the panel, except for live/dead marker, CD10, CD15, CD38 and IgD. An equal number of cells per group (HD vs AA) was included.

References

1. van der Burg M, Kalina T, Perez-Andres M, Vlkova M, Lopez-Granados E, Blanco E, et al. The EuroFlow PID Orientation Tube for Flow Cytometric Diagnostic Screening of Primary Immunodeficiencies of the Lymphoid System. Frontiers in Immunology. 2019;10.

2. Driessen GJ, Dalm VA, van Hagen PM, Grashoff HA, Hartwig NG, van Rossum AM, et al. Common variable immunodeficiency and idiopathic primary hypogammaglobulinemia: two different conditions within the same disease spectrum. Haematologica. 2013;98(10):1617-23.
